# Restoring Endothelial Mitochondrial Homeostasis With a Bioactive Nanoshield Promotes Vascular Regeneration on Cardiovascular Implants

**DOI:** 10.1002/advs.77638

**Published:** 2026-10-25

**Authors:** Xiyu Liu, Wen Zhang, Lunxin Liu, Lan Gui, Yumei Qin, Xiaohui Yang, Longjian Zhang, Chaohua Wang, Jinpeng Hu, Gaoyang Guo, Qing Jiang, Jin Wang, Li Yang, Bo Zhang, Yunbing Wang

**Affiliations:** ^1^ National Engineering Research Center for Biomaterials Sichuan University Chengdu Sichuan China; ^2^ Department of Neurosurgery West China Hospital of Sichuan University Chengdu Sichuan China; ^3^ Key Lab of Advanced Technology of Materials of Education Ministry Southwest Jiaotong University Chengdu Sichuan China

**Keywords:** endothelial regeneration, mitochondrial homeostasis, neointimal hyperplasia, PI3K/AKT/eNOS signaling, vascular regeneration

## Abstract

Defective endothelial regeneration constitutes a fundamental barrier to durable vascular repair. By integrating single cell RNA‐sequencing data from rat carotid balloon injury with ultrastructural and histological evidence from rabbit stented arteries, we identified mitochondrial disorganization and dysfunction as common features of injured endothelial cells. Guided by these findings, we developed a poly(citrate‐polyglycol‐procyanidin) (PCGP) nanoshield to couple mitochondrial protection with anticoagulant and immunoregulatory functions for cardiovascular devices. PCGP preserved mitochondrial integrity, restored endothelial metabolic homeostasis, and rescued the regenerative phenotype of endothelial cells under oxidative stress. In rabbit arterial stent models, this intervention reactivated the PI3K/AKT/eNOS‐nitric oxide axis, accelerated functional re‐endothelialization, and markedly suppressed neointimal hyperplasia. Collectively, these findings support restoration of endothelial mitochondrial homeostasis as an important mechanistic axis associated with vascular repair.

## Graphical abstract

A bioactive poly(citrate‐polyglycol‐procyanidin) (PCGP) nanoshield is developed for cardiovascular implants to restore endothelial mitochondrial homeostasis following vascular injury. By preserving mitochondrial integrity, re‐establishing metabolic balance, and activating PI3K/Akt/eNOS‐NO signaling, the PCGP interface promotes functional endothelial regeneration and supports durable vascular repair.

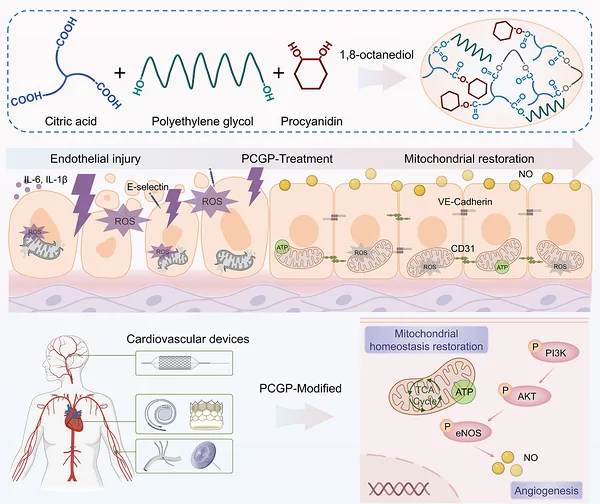

## 1 Introduction

Cardiovascular devices have transformed the treatment of vascular disease, yet their long‐term performance remains constrained by defective endothelial regeneration [1, 2]. Loss of endothelial integrity after implantation initiates thrombosis, chronic inflammation, and maladaptive remodeling, culminating in device failure [3]. Re‐establishing a stable and functional endothelial barrier thus represents a fundamental bottleneck in vascular repair [4].

Mitochondria have emerged as integrators of metabolic and inflammatory signals that shape tissue repair and immune adaptation [5, 6, 7]. In the injured heart, mitochondrial integrity governs cardiomyocyte survival and regenerative capacity, while in T cells, mitochondrial metabolism dictates activation, differentiation, and functional persistence [8, 9]. These findings position mitochondrial homeostasis as an important determinant of cellular recovery across organ systems. In endothelial cells, mitochondria remain important signaling and stress‐adaptive organelles that regulate reactive oxygen species (ROS) signaling, redox balance, Ca^2^^+^ handling, apoptosis, nitric oxide (NO)‐related signaling, and cellular adaptation to injury [10]. Disruption of endothelial mitochondrial homeostasis has been implicated in the development and progression of multiple cardiovascular disorders, including hypertension, atherosclerosis, and vascular aging [11, 12]. Mitochondrial homeostasis may therefore represent a pivotal, yet insufficiently explored, therapeutic axis in restoring vascular integrity after device implantation.

Here, we investigate restoration of endothelial mitochondrial homeostasis as an important mechanistic axis associated with endothelial regeneration after cardiovascular implantation. To therapeutically target this axis, we develop a fully biocompatible poly(citrate‐polyglycol‐procyanidin) (PCGP) nanointerface designed to stabilize mitochondrial function and reprogram endothelial metabolism. Citrate, a central metabolite of the tricarboxylic acid cycle, contributes to metabolic homeostasis and has recently been shown to restore endothelial mitochondrial respiration through AMPK‐related signaling [13]. Procyanidins preserve mitochondrial membrane potential and rebalance mitochondrial quality‐control pathways under oxidative stress [14, 15]. Beyond their mitochondrial effects, citrate provides anticoagulant potential through Ca^2^^+^ chelation, whereas procyanidins confer antioxidant and immunomodulatory activity, collectively creating a microenvironment conducive to endothelial recovery [16]. PCGP preserved mitochondrial integrity, restored endothelial metabolic homeostasis, and rescued the regenerative phenotype of endothelial cells under oxidative stress. In rabbit arterial stent models, this intervention reactivated the PI3K/AKT/eNOS‐NO axis, accelerated functional re‐endothelialization, and markedly suppressed neointimal hyperplasia [17, 18, 19]. Collectively, these findings establish restoration of endothelial mitochondrial homeostasis as a therapeutic strategy for promoting vascular regeneration after cardiovascular implantation.

## 2 Results

### 2.1 Vascular Intervention Disrupts Endothelial Mitochondrial Homeostasis

The mechanical injury caused by balloon dilation and stent strut deployment leads to immediate endothelial denudation. This physical disruption exposes the subendothelial matrix, triggering platelet adhesion and activation, inflammatory cell infiltration, and a burst of ROS production [2]. These secondary pathological events further damage the residual endothelial cells, leading to mitochondrial dysfunction, reduced NO bioavailability, and delayed endothelial repair. We first reanalyzed the publicly available single‐cell RNA sequencing dataset GSE174098, which was obtained from a rat carotid artery balloon injury model. In this dataset, normal carotid arteries were used as the control group, whereas balloon‐injured stenotic carotid arteries were defined as the injury group (Figure 1A). After quality control, all cells were classified into 13 clusters and further annotated into five major vascular cell populations, including vascular smooth muscle cells (VSMCs) (*Acta2, Tagln*, *Cald1* and *Myl9*), fibroblasts (*Col1a1*, *Col3a1*, *Dcn* and *Pdgfra*), endothelial cells (ECs) (*Cdh5*, *Cldn5* and *Sox17*), transitional cells, and macrophages (*Cd68* and *Ncf4*). (Figure 1B and Figure S1A–D). This annotation strategy was consistent with the original description of the article [20].

**FIGURE 1:**
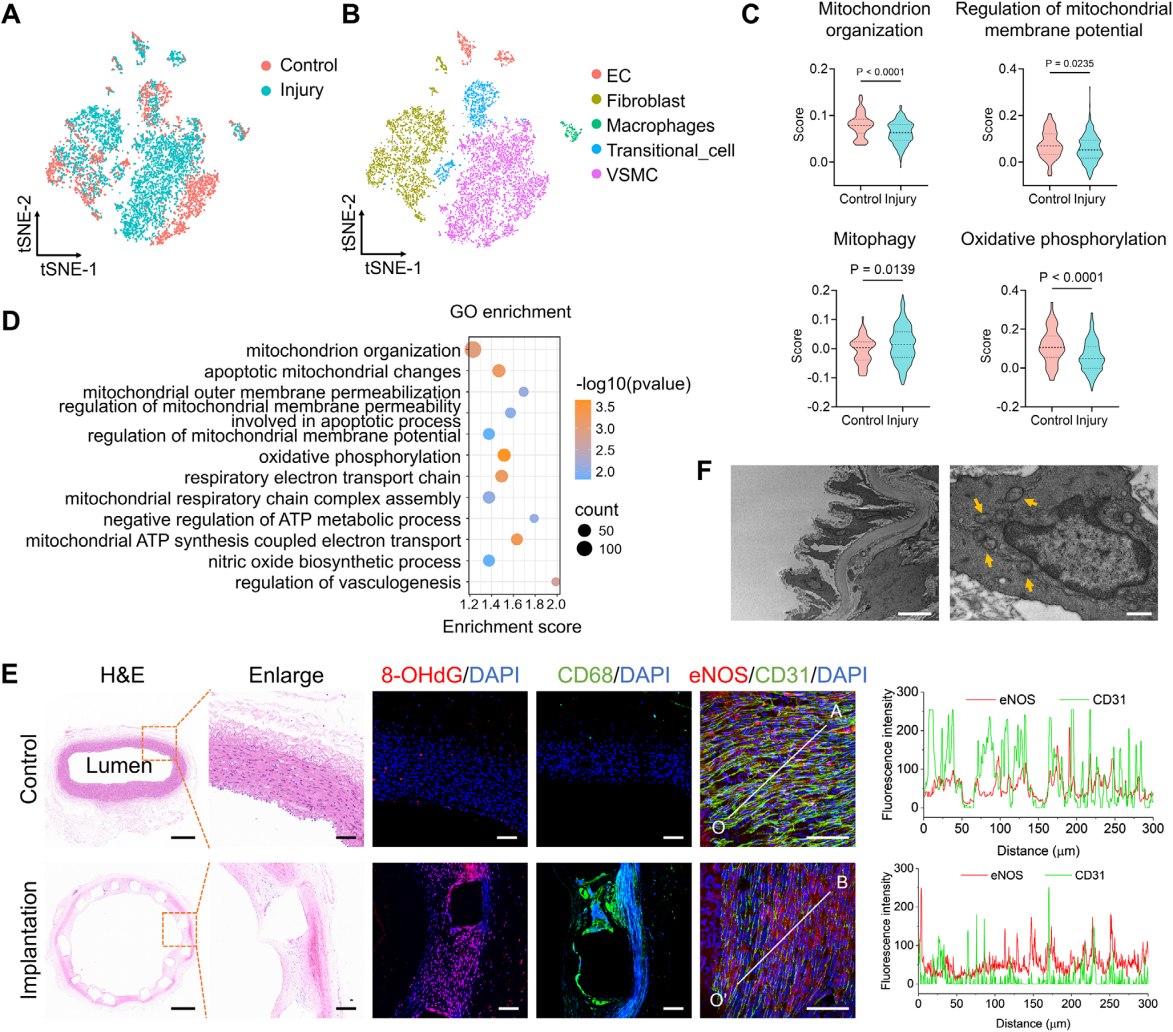
Vascular intervention disrupts endothelial mitochondrial homeostasis. (A) t‐SNE plot showing the distribution of single cells from the control and injury groups. (B) t‐SNE plot showing the annotation of major vascular cell populations. (C) Violin plots showing endothelial cell gene‐set scores. Cell‐level distributional differences were assessed using the Wilcoxon rank‐sum test. (D) Gene Ontology (GO) enrichment analysis of differentially expressed genes in endothelial cells between the injury and control groups. (E) H&E staining and immunofluorescence staining of normal or implanted vessels. 8‐OHdG (red) indicates oxidative DNA damage, CD68 (green) indicates macrophage infiltration, and nuclei are counterstained with DAPI (blue). Confocal laser scanning microscopy (CLSM) images of endothelial on the luminal surfaces. The fluorescence intensities of different cell components along the line segments (OA and O'B) in the images are shown (red: eNOS, green: CD31). Scale bars, 500 µm or 100 µm. (F) Transmission electron microscopy (TEM) images depicting endothelial injury and mitochondrial damage after stent implantation. The yellow arrows indicate damaged mitochondria characterized by swelling, abnormal morphology, and disrupted cristae. Scale bars, 5 µm (left), 500 nm (right).

As endothelial cells are the primary mediators of vascular repair, we next investigated whether vascular injury perturbs endothelial mitochondrial homeostasis. Gene‐set scoring showed that ECs from the injury library exhibited lower cell‐level score distributions for mitochondrion organization, regulation of mitochondrial membrane potential, and oxidative phosphorylation, together with a higher mitophagy score distribution, than those from the control library (Figure 1C). These findings suggest that endothelial cells undergo impaired mitochondrial homeostasis and compensatory mitochondrial quality‐control activation after balloon injury. Functional enrichment analysis based on Gene Ontology (GO) and Kyoto Encyclopedia of Genes and Genomes (KEGG) databases identified pathways closely related to mitochondrial injury and endothelial dysfunction, including apoptotic mitochondrial changes, negative regulation of ATP metabolic process, NO biosynthetic process, regulation of vasculogenesis, VEGF signaling, and cellular senescence (Figure 1D and Figure S1E). Gene set enrichment analysis (GSEA) produced patterns consistent with these observations, with mitochondrial DNA replication and mitochondrial respiratory chain complex I assembly preferentially associated with the control group, whereas cytochrome c release from mitochondria and execution‐phase apoptosis were associated with the injury group (Figure S1F). Together, these exploratory single‐cell transcriptomic patterns suggest a shift from a mitochondria‐supported endothelial phenotype toward a dysfunctional state characterized by impaired energy metabolism and mitochondria‐associated apoptotic signaling.

To further validate these bioinformatic findings in a vascular implant‐related endothelial injury setting, we established a rabbit abdominal aortic stent implantation model. These pathological changes were corroborated by histological and immunofluorescence analyses (Figure 1E). Normal vessels exhibit an intact lumen with well‐aligned endothelial cells, negligible oxidative stress, and minimal inflammatory cell infiltration, as evidenced by low 8‐OHdG and CD68 signals, together with continuous, organized eNOS and CD31 expression. In contrast, weakened and discontinuous eNOS and CD31 staining was observed following stent implantation, accompanied by increased oxidative stress and inflammation, indicative of a severe inflammatory microenvironment and endothelial dysfunction. Transmission electron microscopy (TEM) further revealed ultrastructural abnormalities following device implantation, including disrupted endothelial continuity, altered cellular morphology, and mitochondrial damage (Figure 1F).

Collectively, the single‐cell transcriptomic analysis and in vivo implantation model consistently demonstrate that vascular intervention provokes endothelial injury, oxidative stress, and mitochondrial dysfunction. These pathological events may jointly compromise endothelial repair and vascular homeostasis, thereby contributing to delayed endothelialization and adverse vascular remodeling. On this basis, we designed a bioactive coating aimed at restoring endothelial mitochondrial homeostasis, reducing oxidative and inflammatory stress, and promoting endothelial regeneration, thereby improving the long‐term biological performance of cardiovascular implants.

### 2.2 Construction and Characterization of PCGP

Given the critical role of mitochondria in maintaining endothelial homeostasis, we developed a poly(citrate‐polyglycol‐procyanidin) (PCGP)‐based nanoshield to counteract the endothelial mitochondrial injury induced by cardiovascular device implantation and the pathological microenvironment. PCGP and poly(citrate‐polyglycol) (PCG) were synthesized through a one‐pot thermal polycondensation reaction (Figure 2A). ^1^H nuclear magnetic resonance (^1^H NMR) spectrum (Figure S2) confirmed representative methylene signals (2.7–3.1 ppm) from citric acid, a characteristic peak at 3.6 ppm from polyethylene glycol, and aromatic peaks (6.5–8.5 ppm) from procyanidin units, verifying successful copolymerization. Attenuated total reflection Fourier transform infrared (ATR‐FTIR) analysis (Figure S3) further identified the stretching vibrations of hydroxyl (3000–3700 cm^−1^), methylene (2800–3000 cm^−1^), and carbonyl (1600–1800 cm^−1^) groups, along with a phenyl ring band at 1520 cm^−1^, consistent with procyanidin incorporation. Together, these spectra confirm the successful synthesis of PCGP with integrated citrate, glycol, and polyphenolic domains (M_n_ ∼ 1155 Da), forming a versatile precursor for surface functionalization.

**FIGURE 2:**
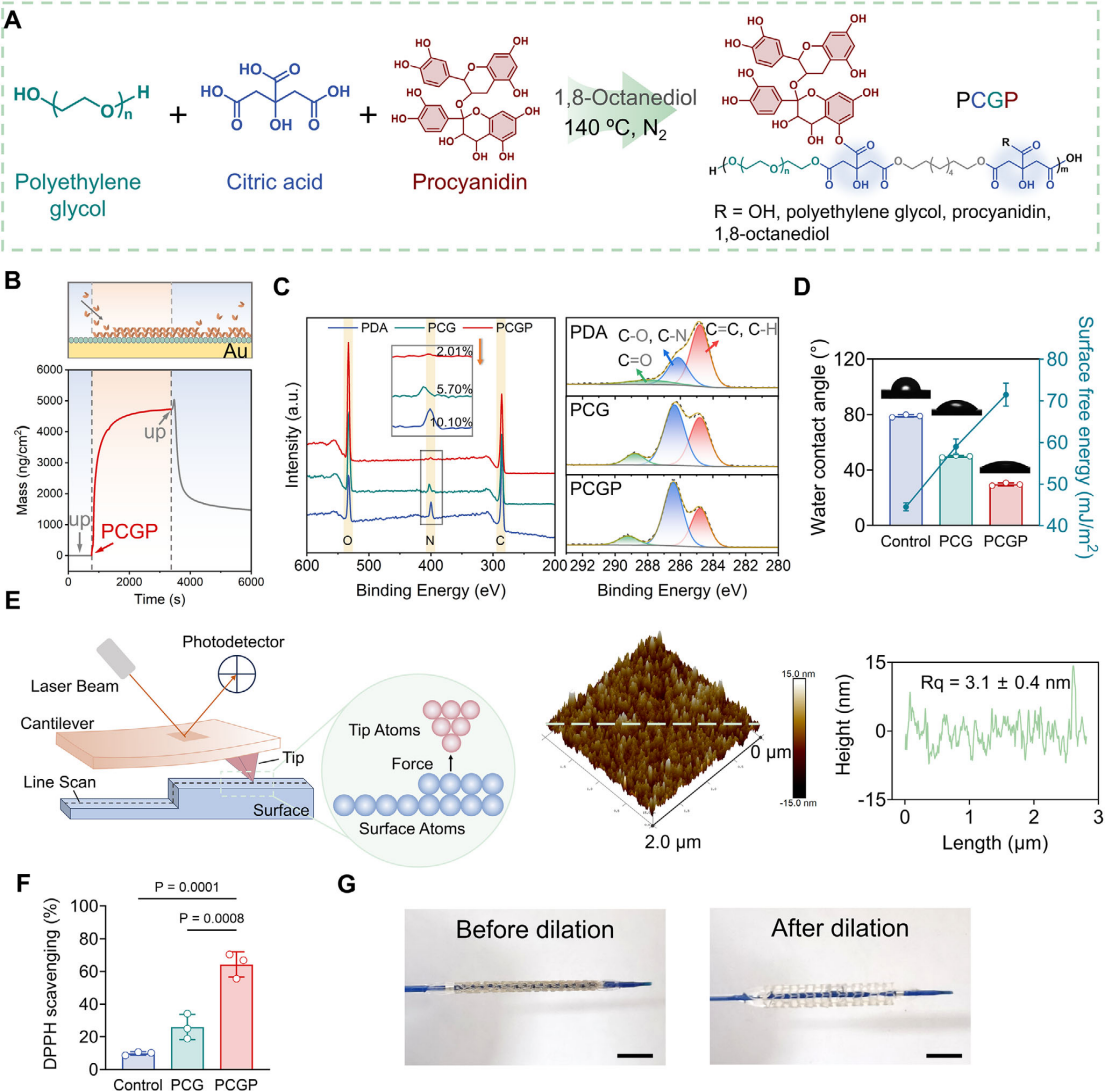
Construction and characterization of PCGP nanoshield. (A) Synthesis of PCGP via a one‐pot thermal polycondensation reaction. (B) Real‐time quartz crystal microbalance with dissipation (QCM‐D) monitoring showing the immobilization of PCGP on PDA‐modified gold substrates. (C) X‐ray photoelectron spectroscopy (XPS) and high‐resolution C1s spectra confirming the incorporation of PCGP with weakened N signals and enhanced C‐O signals. (D) Contact angle measurements revealing enhanced hydrophilicity and surface free energy of PCGP interfaces. (E) Atomic force microscopy (AFM) illustrating the working principle and nanoscale surface roughness of PCGP coatings. (F) Free‐radical scavenging performance evaluated by the DPPH assay, demonstrating the superior antioxidant capacity of PCGP. (G) Photographs of PCGP stents before and after balloon dilation in PBS at 37°C, showing the intact coating integrity. Scale bar, 1 cm. Data are presented as mean ± standard deviation (*n* = 3). Statistical significance was assessed by one‐way ANOVA with Tukey's multiple comparisons test.

The PCGP nanoshield was fabricated through a sequential surface‐assembly strategy. Bare stents were first functionalized with a classic mussel‐inspired amine‐rich polydopamine (PDA) layer formed by the co‐deposition of dopamine and 1,6‐hexanediamine [21]. PCGP was then covalently anchored using 1‐ethyl‐3(3‐dimethyl aminopropyl) carbodiimide (EDC) and N‐hydroxysuccinimide (NHS). Quartz crystal microbalance with dissipation (QCM‐D) further verified mass loading and PCGP layer formation during assembly (Figure 2B). X‐ray photoelectron spectroscopy (XPS) confirmed the successful incorporation of PCGP, with characteristic C, O, and N signals, as well as a pronounced increase in oxygen content (Figure 2C and Table S1). High‐resolution C1s spectra exhibited an intensified C‐O component, consistent with the introduction of citrate and procyanidin moieties. The resulting PCGP interface exhibited markedly enhanced hydrophilicity and surface free energy compared with bare substrates, which may facilitate endothelial cell adhesion and spreading [22] (Figure 2D). Atomic force microscopy (AFM) revealed a nanoscale topographic transformation from the smooth substrate (1.0 ± 0.2 nm) to a textured PCGP surface (3.1 ± 0.4 nm; Figure 2E and Figure S4), while ellipsometry measured an average film thickness of ∼ 88 nm. The DPPH test confirmed the coating had a strong antioxidant capacity, achieving a 64% radical clearance within 1 h (Figure 2F). To evaluate the long‐term stability of coatings under physiological flow‐mimicking conditions, PDA‐ and PCGP‐coated samples were subjected to continuous PBS circulation for up to 28 days (Figure S5A). XPS analysis showed that the surface N content of the PCGP group remained markedly lower than that of the corresponding PDA group throughout the experiment, indicating that the PCGP layer was not completely removed after 28 days of dynamic circulation (Figure S5B). Citrate was continuously released from the coating (Figure S6). When applied to vascular stents, PCGP layer maintained its integrity after simulated balloon dilation in PBS at 37°C, with scanning electron microscopy (SEM) revealing a continuous nanostructured morphology (Figure 2G and Figure S7).

### 2.3 PCGP Restores Endothelial Mitochondrial Homeostasis and Rebalances Metabolism In Vitro

Keeping the balance between ROS generation and scavenging is critical for endothelial redox homeostasis [23]. Under oxidative or inflammatory stress, excessive mitochondrial ROS (mtROS) accumulation disrupts this equilibrium, leading to endothelial dysfunction—a key contributor to cardiovascular diseases and implant failure [24].

To assess whether PCGP modulates oxidative stress in endothelial cells, we first quantified intracellular ROS levels using DCFH‐DA and DHE probes (Figure S8). Exposure of human umbilical vein endothelial cells (HUVECs) to 400 µM H_2_O_2_ markedly increased ROS fluorescence intensity compared to untreated controls, confirming oxidative damage (Figure 3A,B). In contrast, cells cultured on PCGP‐coated substrates displayed notably reduced DCFH‐DA and DHE signals, demonstrating that the incorporation of procyanidin significantly enhances the antioxidant capacity of the coating. Consistently, quantitative real‐time polymerase chain reaction (qRT‐PCR) analysis revealed that oxidative stress upregulated antioxidant defense genes (*SOD2*, *PRDX3*, and *NQO1*), reflecting redox system disruption, whereas PCGP treatment maintained their expression near baseline, indicating preserved antioxidant homeostasis [25] (Figure S9).

**FIGURE 3:**
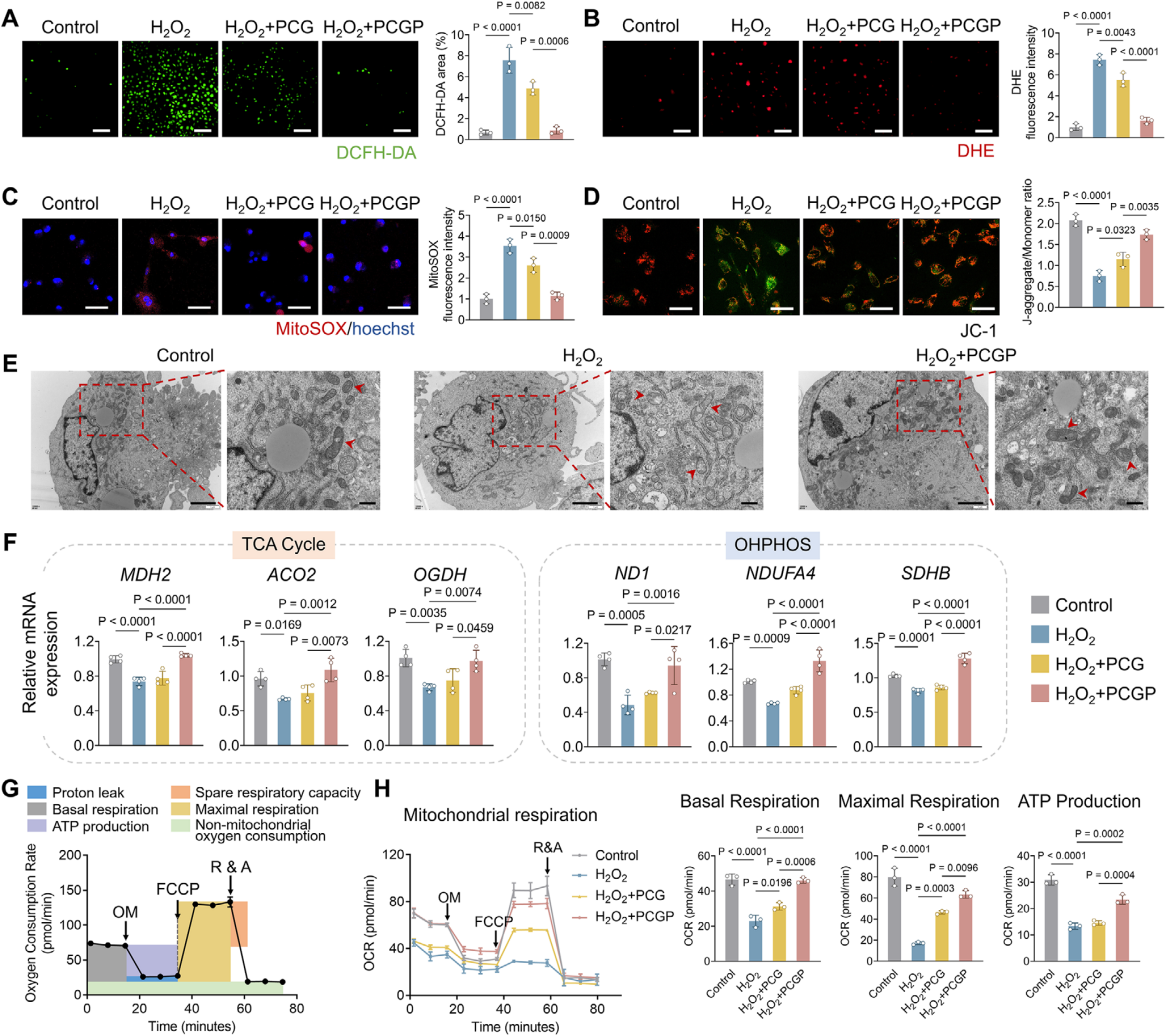
In vitro evaluation of mitochondrial homeostasis and endothelial metabolism. (A) Fluorescence imaging and quantitative analysis of intracellular ROS using DCFH‐DA. Scale bar, 200 µm. (B) Detection and quantification of superoxide anion generation using DHE staining. Scale bar, 200 µm. (C) MitoSOX fluorescence and corresponding quantification showing reduced mitochondrial ROS levels in PCGP‐treated cells. Scale bar, 50 µm. (D) JC‐1 staining and quantification demonstrating preservation of mitochondrial membrane potential. Scale bar, 50 µm. (E) Transmission electron microscopy (TEM) images showing ultrastructural preservation of mitochondria in the PCGP group compared to oxidative damage in H_2_O_2_‐treated cells. Red arrowheads indicate mitochondria. Scale bar, 1 µm. (F) Quantitative real‐time polymerase chain reaction (qRT‐PCR) analysis of tricarboxylic acid (TCA) cycle genes (*MDH2*, *ACO2*, and *OGDH*) and oxidative phosphorylation (OXPHOS) genes (*ND1*, *NDUFA4*, and *SDHB*), indicating recovery of mitochondrial metabolic gene expression (*n* = 4). (G) Schematic of Seahorse analysis with specific mitochondrial respiration parameters. Abbreviations: OM, oligomycin; FCCP, carbonyl cyanide‐p‐trifluoromethoxyphenyl hydrazone; R&A, rotenone and antimycin A. (H) Seahorse extracellular flux analysis using the Cell Mito Stress Test (Agilent) showing basal respiration, maximal respiration, and ATP production. Data are presented as mean ± standard deviation (*n* = 3). Statistical significance was assessed by one‐way ANOVA with Tukey's multiple comparisons test.

We next examined whether PCGP preserves mitochondrial integrity. MitoSOX staining revealed elevated mtROS in H_2_O_2_‐treated HUVECs, which was markedly reduced in the PCGP group (Figure 3C). The mitochondrial transmembrane potential (MMP) is critical for maintaining both mitochondrial function and cellular homeostasis [26]. JC‐1 fluorescence imaging showed that H_2_O_2_ exposure depolarized the mitochondrial membrane, yielding predominant green fluorescence from JC‐1 monomers, whereas PCGP maintained high mitochondrial membrane potential with strong red aggregate fluorescence [27] (Figure 3D). TEM further confirmed that PCGP prevented oxidative damage‐induced mitochondrial swelling and cristae loss [28] (Figure 3E). These results indicate that the PCGP nanoshield protects mitochondrial structure and electrochemical potential by limiting mtROS overproduction.

Mitochondrial homeostasis is intimately linked to endothelial metabolism and function [29]. We analyzed the expression of key metabolic and respiratory genes. PCGP upregulated tricarboxylic acid (TCA) cycle‐related genes (*MDH2*, *ACO2*, and *OGDH*) and oxidative phosphorylation (OXPHOS) genes (*ND1*, *NDUFA4*, and *SDHB*) [30, 31](Figure 3F). We also measured intracellular citrate levels in HUVECs (Figure S10). H_2_O_2_ exposure reduced the intracellular citrate pool, whereas both PCG and PCGP treatment partially restored intracellular citrate levels. To further determine whether PCGP restores endothelial bioenergetic function under oxidative stress, we performed Seahorse extracellular flux analysis to evaluate mitochondrial respiration and glycolytic metabolism (Figure 3G). H_2_O_2_ exposure markedly decreased the oxygen consumption rate (OCR), accompanied by substantial reductions in basal respiration, maximal respiration, and ATP‐linked respiration (Figure 3H). Although PCG partially rescued these parameters, PCGP elicited a more robust recovery. H_2_O_2_ treatment also suppressed the extracellular acidification rate and glycolytic proton efflux rate, suggesting a concurrent impairment of glycolytic metabolism (Figure S11). PCGP significantly restored glycolytic activity. These findings demonstrate that PCGP does not merely enhance a single metabolic pathway but broadly reverses oxidative stress‐induced bioenergetic collapse, thereby restoring coordinated mitochondrial and glycolytic homeostasis in endothelial cells.

Together, these findings demonstrate that PCGP nanoshield re‐establishes mitochondrial homeostasis in endothelial cells by scavenging mtROS, stabilizing membrane potential, preserving ultrastructure, and sustaining metabolic function.

### 2.4 PCGP Restores Endothelial Function In Vitro

Mitochondrial damage can trigger apoptosis. H_2_O_2_‐treated cells exhibited markedly higher early and late apoptotic fractions, whereas PCGP treatment reduced total apoptosis to ∼ 5.21%, comparable to control levels (Figure 4A and Figure S12).

**FIGURE 4:**
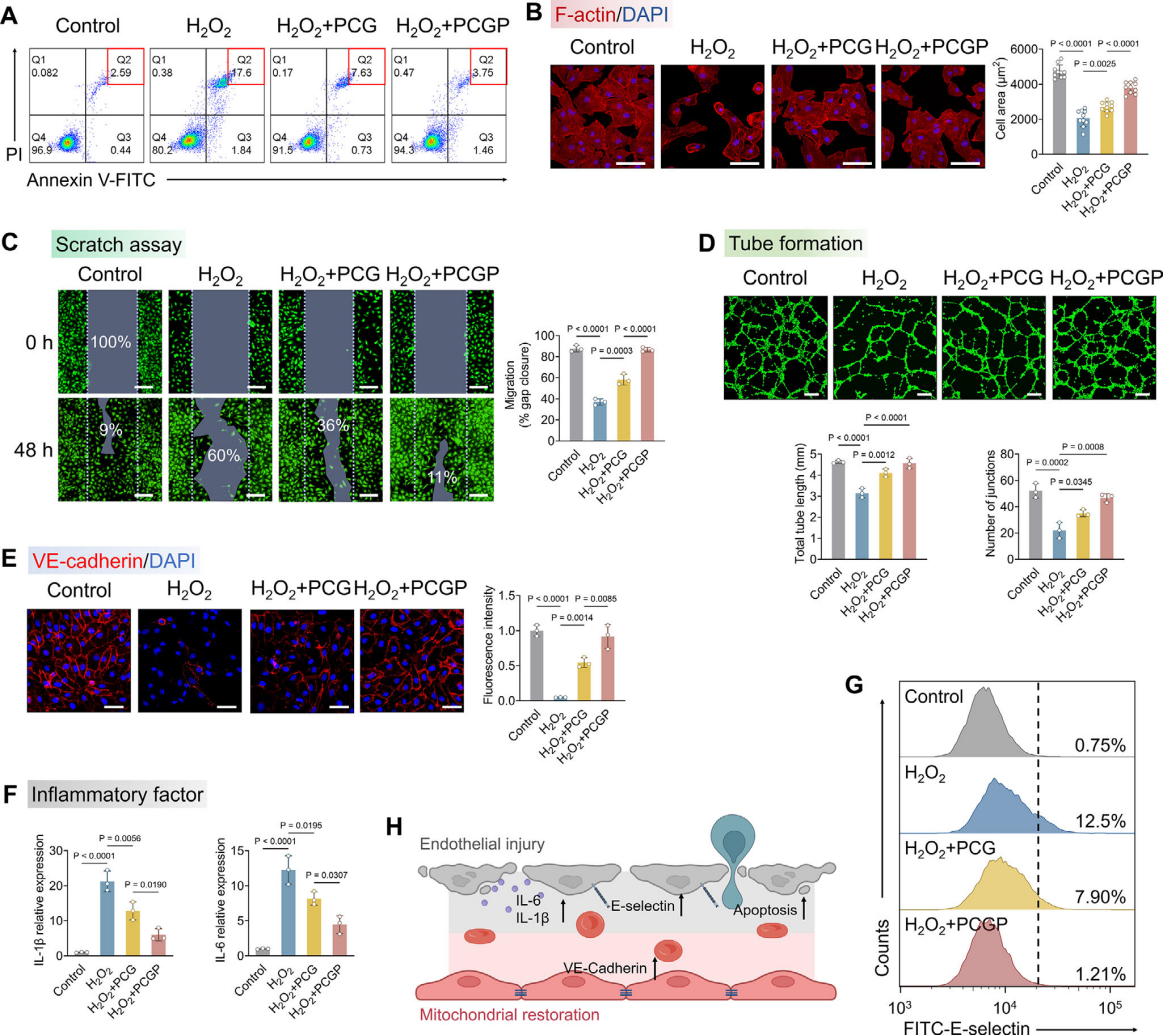
PCGP restores endothelial function in vitro. (A) Flow cytometric analysis of apoptosis using Annexin V‐FITC/PI staining. (B) TRITC‐phalloidin staining with quantitative analysis of HUVEC cytoskeletal organization under H_2_O_2_‐induced stress. Scale bar, 100 µm (n = 10). (C) Fluorescence images and quantification of HUVECs migration under oxidative conditions. Scale bar, 200 µm. (D) Tube formation assay on Matrigel for 8 h showing representative fluorescence images and quantification of junction number and total tube length. Scale bar, 200 µm. (E) VE‐cadherin immunostaining and quantitative analysis of adherens junction integrity in HUVECs cultured on different coatings under H_2_O_2_ challenge. Scale bar, 50 µm. (F) Quantitative real‐time polymerase chain reaction (qRT‐PCR) assay of pro‐inflammatory factors (IL‐1β and IL‐6) showing reduced endothelial inflammation. (G) Flow cytometric analysis of E‐selectin expression showing reduced endothelial activation on PCGP surfaces. (H) PCGP nanoshield reinforces functional homeostasis in endothelial cells under oxidative stress, maintaining barrier integrity and promoting anti‐inflammatory functions. Unless otherwise indicated, data are presented as mean ± standard deviation (n = 3). Statistical significance was assessed by one‐way ANOVA with Tukey's multiple comparisons test. Panel H was created with BioRender.com.

Maintaining mitochondrial homeostasis is essential for preserving endothelial functions, including angiogenesis, barrier integrity, and anti‐inflammatory responses. Morphologically, PCGP prevented H_2_O_2_‐induced cytoskeletal collapse. HUVECs cultured on PCGP‐coated surfaces maintained well‐spread morphologies with organized actin filaments, in contrast to the contracted and fragmented cytoskeleton observed in the H_2_O_2_ group (Figure 4B). PCGP promoted cell migration under oxidative conditions, achieving 89% wound closure after 48 h (Figure 4C), and enhanced tube formation, reflected by increased junction numbers and total tube length (Figure 4D). These results collectively demonstrate that PCGP supports cytoskeletal stability and angiogenic potential—key determinants of rapid endothelial recovery after implantation. Additionally, PCGP preserved endothelial junction integrity. VE‐cadherin staining in the PCGP group was strong, continuous, and circumferential, while oxidative stress disrupted these adherens junctions [32] (Figure 4E). Furthermore, PCGP significantly suppressed endothelial activation, as evidenced by downregulated E‐selectin expression and reduced gene expression of key pro‐inflammatory factors (IL‐1β and IL‐6) (Figure 4F,G). Consequently, it would be expected to reduce leukocyte adhesion [33, 34]. Together, these findings demonstrate that PCGP nanoshield reinforces both structural and functional homeostasis in endothelial cells under oxidative stress, thereby promoting angiogenic, migratory, barrier‐protective, and anti‐inflammatory functions essential for vascular regeneration (Figure 4H).

To further determine whether mitochondrial dysfunction directly impairs endothelial regenerative capacity, HUVECs were exposed to rotenone, an inhibitor of mitochondrial complex I. Rotenone treatment markedly reduced cell viability and severely impaired endothelial tube formation, as evidenced by decreases in total tube length and the number of junctions (Figure S13A,B). Consistently, rotenone disrupted endothelial cell‐cell contacts and substantially decreased VE‐cadherin expression (Figure S13C). PCG partially alleviated these impairments, whereas PCGP produced a more pronounced restorative effect, improving cell viability, recovering network formation, and preserving VE‐cadherin‐positive intercellular junctions. These findings indicate that mitochondrial respiratory dysfunction is sufficient to compromise the survival, angiogenic activity, and barrier integrity of endothelial cells, while PCGP effectively rescues the endothelial regenerative phenotype under mitochondria‐specific stress.

### 2.5 PCGP Promotes Vascular Healing Through Activation of the PI3K/AKT/eNOS Pathway

Building upon the excellent biological functions of PCGP, we evaluated the in vivo performance of PCGP‐coated stents (PCGPs) using a rabbit abdominal aortic stent implantation model (Figure 5A). Photon‐counting CT confirmed accurate stent deployment and patency (Figure 5B). To elucidate the molecular mechanisms underlying the vascular healing effects of PCGP, we performed RNA sequencing on peri‐stent arterial tissue at 3 weeks post‐implantation, using unmodified PLA stents as controls. Differentially expressed genes (DEGs) analysis revealed 334 significantly modulated genes (fold change > 1.5 and p < 0.05, Figure 5C). Notably, multiple genes linked to mitochondrial metabolism were upregulated in the PCGP group (Figure 5D). GO enrichment revealed that these genes were primarily enriched in the regulation of mitochondrial homeostasis, endothelial function, and angiogenesis (Figure 5E).

**FIGURE 5:**
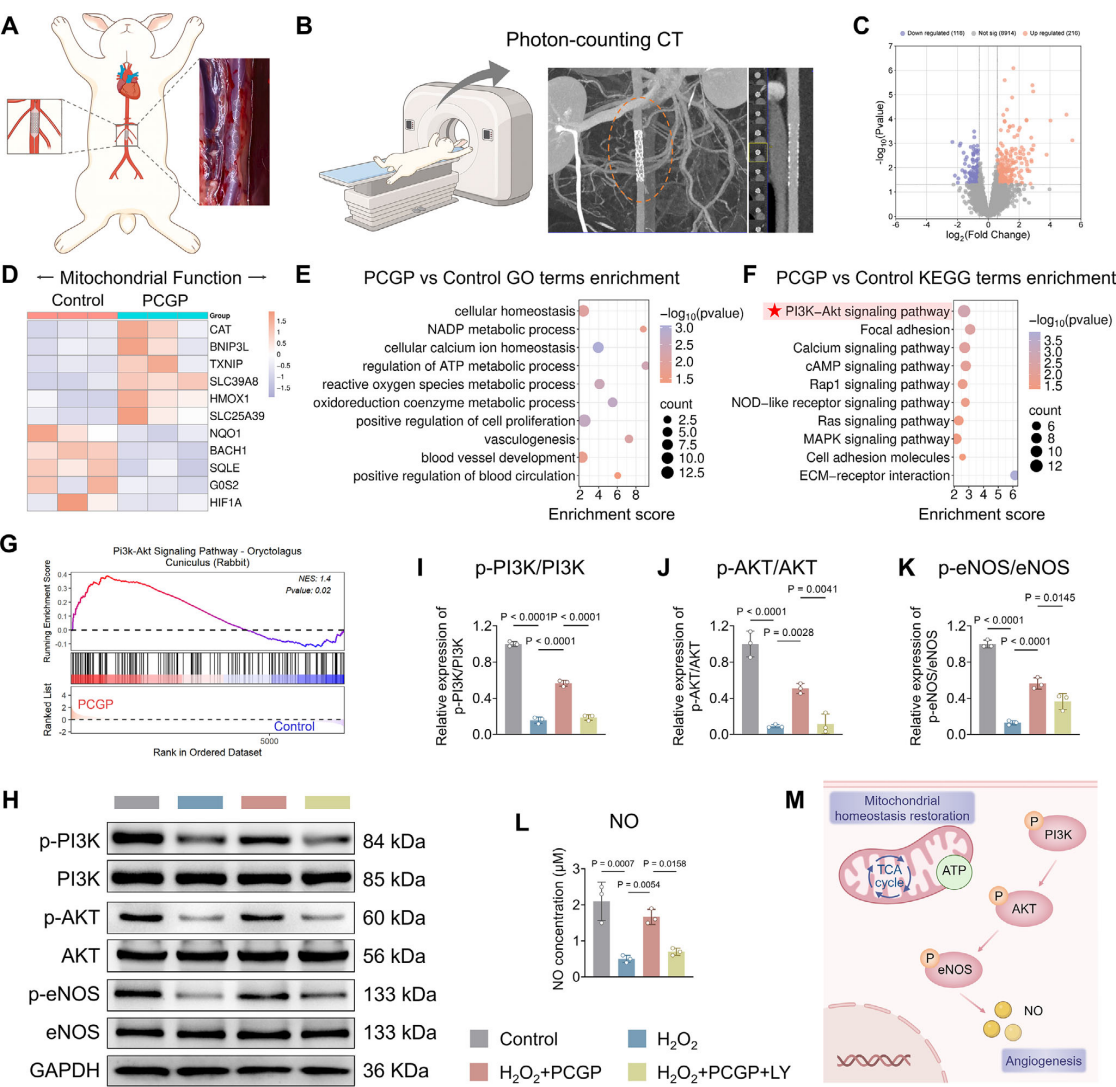
PCGP promotes vascular healing through activation of the PI3K/AKT/eNOS pathway. (A) Experimental illustration of abdominal aortic stent implantation. (B) Photon‐counting CT images confirming accurate positioning and patency of stents. (C) Volcano plot displaying differentially expressed genes (DEGs) between PCGP stents and bare PLA stents (fold change > 1.5 and *p* < 0.05). (D) Heatmap of mitochondrial function‐related DEGs illustrating the restoration of mitochondrial activity in the PCGP group. (E) Gene Ontology (GO) enrichment showing DEGs associated with mitochondrial homeostasis, endothelial cell functions and angiogenesis. (F) Kyoto Encyclopedia of Genes and Genomes (KEGG) pathway enrichment highlighting the regulation of vascular healing‐related pathways. (G) Gene set enrichment analysis (GSEA) demonstrating positive regulation of the PI3K‐AKT signaling pathway. (H‐K) Western blot and semiquantitative analysis confirming increased phosphorylation of PI3K, AKT, and eNOS in the PCGP group. Abbreviations: LY, LY294002. (L) Nitric oxide (NO) release in different groups. (M) Schematic illustration summarizing PCGP‐mediated restoration of mitochondrial homeostasis and activation of the PI3K/AKT/eNOS axis supporting endothelial recovery and angiogenic function. Data are presented as mean ± standard deviation (*n*  =  3). Statistical significance was assessed by one‐way ANOVA with Tukey's multiple comparisons test. ns, no significance. Panel M created with BioRender.com.

KEGG pathway analysis (Figure 5F) highlighted several signaling cascades relevant to vascular remodeling, including ECM‐receptor interaction, focal adhesion, PI3K‐Akt signaling, MAPK signaling, and calcium signaling pathways. GSEA further confirmed significant activation of PI3K‐Akt signaling pathways in the PCGP group (Figure 5G). Western blot analysis validated the activation of the PI3K/AKT/eNOS signaling axis (Figure 5H and Figure S14). The phosphorylated ratios of PI3K, AKT, and eNOS were markedly elevated in PCGP‐treated HUVECs compared to the H_2_O_2_ group, accompanied by significantly higher NO production (Figure 5I–L). These effects were substantially attenuated by LY294002, a broad‐spectrum PI3K inhibitor. These findings associate PCGP‐mediated endothelial recovery with restoration of mitochondrial homeostasis and activation of the PI3K/AKT/eNOS–NO signaling axis (Figure 5M).

### 2.6 PCGP Exhibits Anti‐Thrombogenic Performance and Potent Immunoregulatory Activity

Cardiovascular materials are typically in contact with blood, necessitating that an ideal biointerface effectively prevent protein adsorption, platelet activation, and fibrin network formation [35]. We first evaluated the anticoagulant performance of the PCGP coating in vitro (Figure S15). The anticoagulant mechanism of citrate derives from calcium chelation, which inhibits the intrinsic coagulation cascade, suppressing thrombin generation and platelet aggregation [36]. XPS analysis confirmed calcium binding on PCG and PCGP coatings after immersion in a CaCl_2_ solution simulating plasma concentration for 1 h (Figure 6A). Fluorescence imaging of FITC‐labeled bovine serum albumin (BSA‐FITC) revealed strong protein adsorption on unmodified surfaces, whereas PCGP showed markedly reduced fluorescence intensity (Figure 6B,C), confirming its potent antifouling capability. When whole blood or platelet‐rich plasma (PRP) was incubated with the samples, control surfaces exhibited extensive platelet and erythrocyte adhesion, accompanied by pseudopodia formation characteristic of platelet activation (Figure S16A,C). In contrast, PCGP‐coated surfaces displayed minimal blood cell adhesion, reducing platelet density by 76% and erythrocyte attachment by 94% relative to bare substrates (Figure S16B,D). The antithrombotic efficacy of PCGP was further validated *ex vivo* using a New Zealand white rabbit arteriovenous shunt model (Figure 6D). After 1 h of circulation, severe occlusion and dense fibrin networks were observed on control grafts, whereas PCGP‐coated surfaces remained fully patent and exhibited a fivefold reduction in thrombus weight (Figure 6E–H).

**FIGURE 6:**
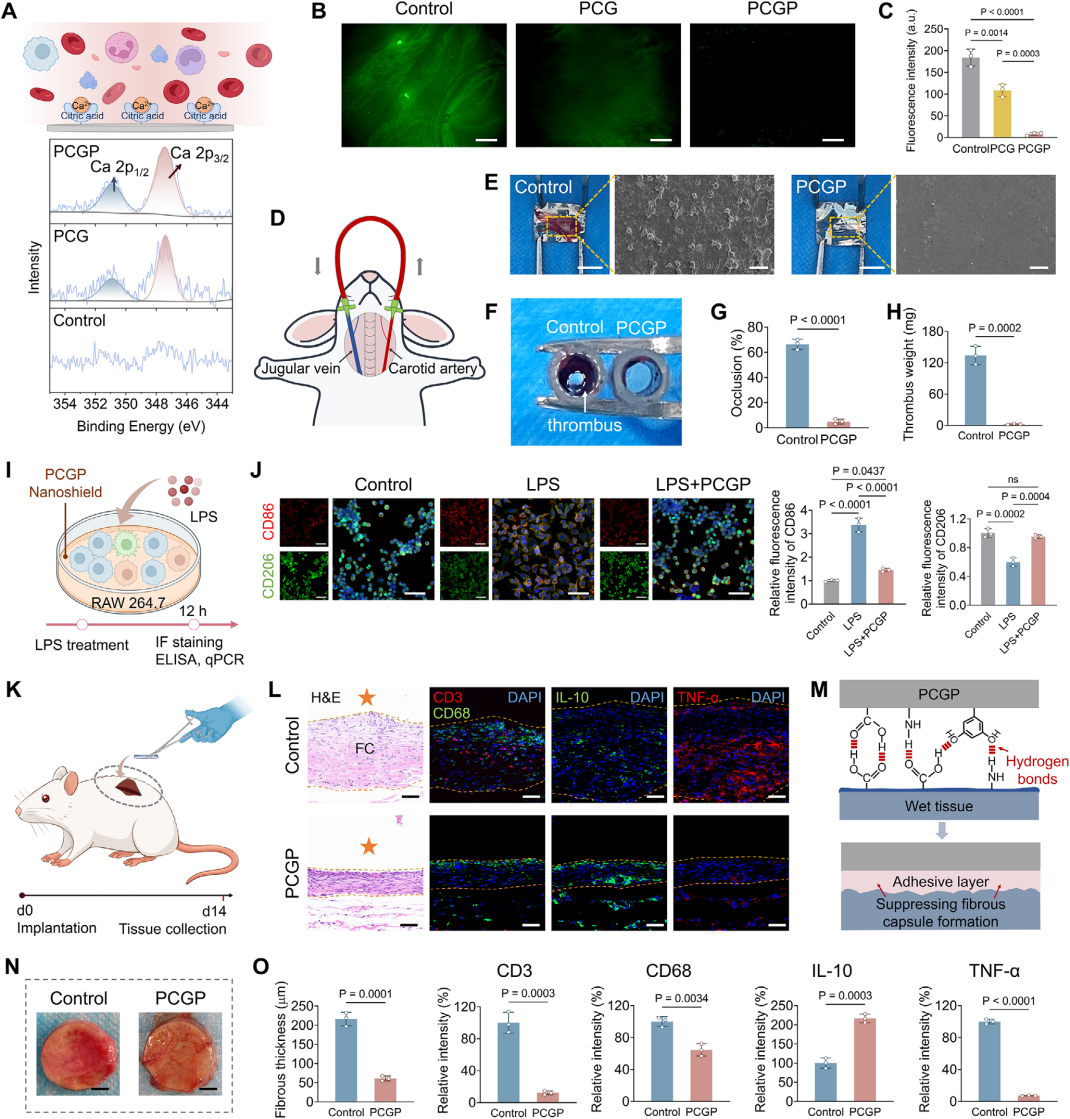
PCGP exhibits anti‐thrombogenic performance and potent immunoregulatory activity. (A) Schematic illustration of the antithrombotic capacity derived from PCGP‐mediated calcium chelation. High‐resolution Ca 2p spectra confirm calcium binding on the material surfaces. (B) Fluorescence images of BSA‐FITC adsorbed on the sample surfaces. Scale bar, 200 µm. (C) Quantitative analysis of BSA‐FITC. (D) Schematic diagram of *ex vivo* arteriovenous shunt assay. (E) Photographs and scanning electron microscopy (SEM) images of thrombi on sample surfaces. Scale bars, 5 mm or 20 µm. (F) Cross‐sectional photographs showing thrombi deposition on different samples after 1 h of circulation. (G,H) Quantitative analysis of occlusion and thrombosis weight. (I) Schematic illustration of the in vitro evaluation of macrophage behavior. (J) Immunofluorescence staining and quantitative analysis of CD86 (M1 marker) and CD206 (M2 marker) in macrophages cultured on different surfaces. Scale bar, 50 µm. (K) Schematic diagram of subcutaneous implantation in rats. (L) Representative histological and immunofluorescence images of implants retrieved at day 14. Nuclei are stained with DAPI (blue); macrophages (CD68) and anti‐inflammatory macrophages (IL‐10) appear green; T cells (CD3) and pro‐inflammatory macrophages (TNF‐α) appear red. The orange dotted line marks the fibrous capsule; implant positions are indicated by asterisks. Scale bars, 50 µm. FC, fibrous capsule. (M) Schematic illustrations of conformal interfacial integration enabled by PCGP, minimizing immune infiltration and fibrotic encapsulation. (N) Representative photographs of materials and surrounding tissues. Scale bar, 3 mm. (O) Quantification of fibrous capsule thickness, CD3 expression, CD68 expression, IL‐10 expression, and TNF‐α expression of the fibrous capsule at the implant‐tissue interface. Data are presented as mean ± standard deviation (*n*  =  3). Statistical significance was assessed by one‐way ANOVA with Tukey's multiple comparisons test (J) or two‐tailed Student's t‐test (G, H, and O). ns, no significance. Panels A and I were created with BioRender.com.

Mechanistically, the exceptional hemocompatibility of PCGP nanoshield arises from three synergistic effects: (1) calcium chelation by citrate that interrupts coagulation initiation; (2) the formation of a hydrated antifouling layer by polyethylene glycol segments that resists protein and platelet adhesion [37]; and (3) electrostatic repulsion between negatively charged procyanidin and fibrinogen that prevents fibrin crosslinking. Collectively, these mechanisms confer antithrombotic protection and minimize post‐implant thrombosis.

Moreover, vascular injury triggers a local inflammatory cascade that delays endothelial recovery and drives fibrosis. Thus, modulating the peri‐implant immune microenvironment is essential for rapid and functional endothelial regeneration [38]. To assess the immunoregulatory capacity of PCGP, we cultured mouse mononuclear macrophage cells (RAW 264.7) on different surfaces in vitro (Figure 6I). On unmodified substrates, macrophages exhibited activated morphologies with extended pseudopodia, whereas those on PCGP remained rounded and quiescent, indicative of excellent biocompatibility (Figure S17). Macrophage polarization was further examined using CD86 and CD206 markers for M1 and M2 phenotypes, respectively [39]. Upon LPS stimulation, macrophages on control surfaces expressed high levels of CD86, while PCGP markedly suppressed CD86 and increased CD206 fluorescence intensity (Figure 6J), indicating a shift toward a reparative M2 phenotype. qRT‐PCR corroborated this trend: PCGP downregulated pro‐inflammatory genes (*Tnf*, *Nos2*) and upregulated anti‐inflammatory markers (*Mrc1*, *Il4*) (Figure S18). ELISA assays further confirmed these findings, showing decreased secretion of TNF‐α and IL‐6 in the PCGP group [40] (Figure S19). Collectively, these results demonstrate that PCGP reprograms macrophages toward an anti‐inflammatory, pro‐regenerative state (Figure S20).

To evaluate the in vivo immune response and tissue integration, we employed a rat subcutaneous implantation model (Figure 6K). After 14 days, optical photographs and H&E staining revealed an extensive fibrous capsule formation around control implants, whereas PCGP‐coated implants exhibited a significantly thinner capsule and attenuated inflammation (Figure 6L,N). Immunofluorescence analysis showed fewer infiltrated macrophages (CD68^+^), T cells (CD3^+^), and pro‐inflammatory TNF‐α^+^ cells, alongside increased anti‐inflammatory IL‐10^+^ macrophages surrounding PCGP‐coated implants (Figure 6O).

The conformal tissue integration afforded by PCGP nanoshield stems from its multifunctional reactive groups (quinone, amino, carboxyl, and hydroxyphenyl moieties) and aromatic backbone, which may promote stable chemical and physical coupling with adjacent tissues [41]. This seamless interface is associated with reduced immune infiltration and fibrotic encapsulation (Figure 6M). By contrast, untreated implants lack such conformal integration, leading to immune infiltration and fibrous capsule formation.

Together, these findings establish PCGP as a multifunctional biointerface that integrates anticoagulant activity, macrophage immunomodulation, and conformal tissue integration, thereby creating a local microenvironment conducive to endothelial regeneration.

### 2.7 Biological Effects of PCGP in Rabbits

In the rabbit abdominal aortic stent implantation model, bare PLA stents and rapamycin‐eluting stents (RAPAs) served as controls [42]. Stented aortas were harvested at 3 weeks and 3 months post‐implantation for histological, immunological, and safety analysis. Photographs show that thick neointima grew on RAPAs, while neointimal formation was much slower or even negligible on PCGPs at 3 months (Figure S21). Histological staining revealed complete stent expansion across all groups but marked differences in neointimal proliferation (Figure 7A). Quantitative analysis demonstrated that PCGPs significantly reduced neointimal area and stenosis ratio at both 3 weeks and 3 months relative to bare stents (Figure 7B,C). RAPAs showed strong short‐term inhibition of neointimal hyperplasia (NIH, 1.33 ± 0.06 mm^2^ and 20.8 ± 0.6% stenosis at 3 weeks), but this effect was unsustained, with NIH rebounding by 3 months (2.13 ± 0.21 mm^2^ and 29.3 ± 0.8%). In contrast, PCGPs exhibited mild NIH progression over the same period (1.53 ± 0.05 mm^2^ and 22.7 ± 1.7% at 3 months), indicating superior long‐term vascular healing.

**FIGURE 7:**
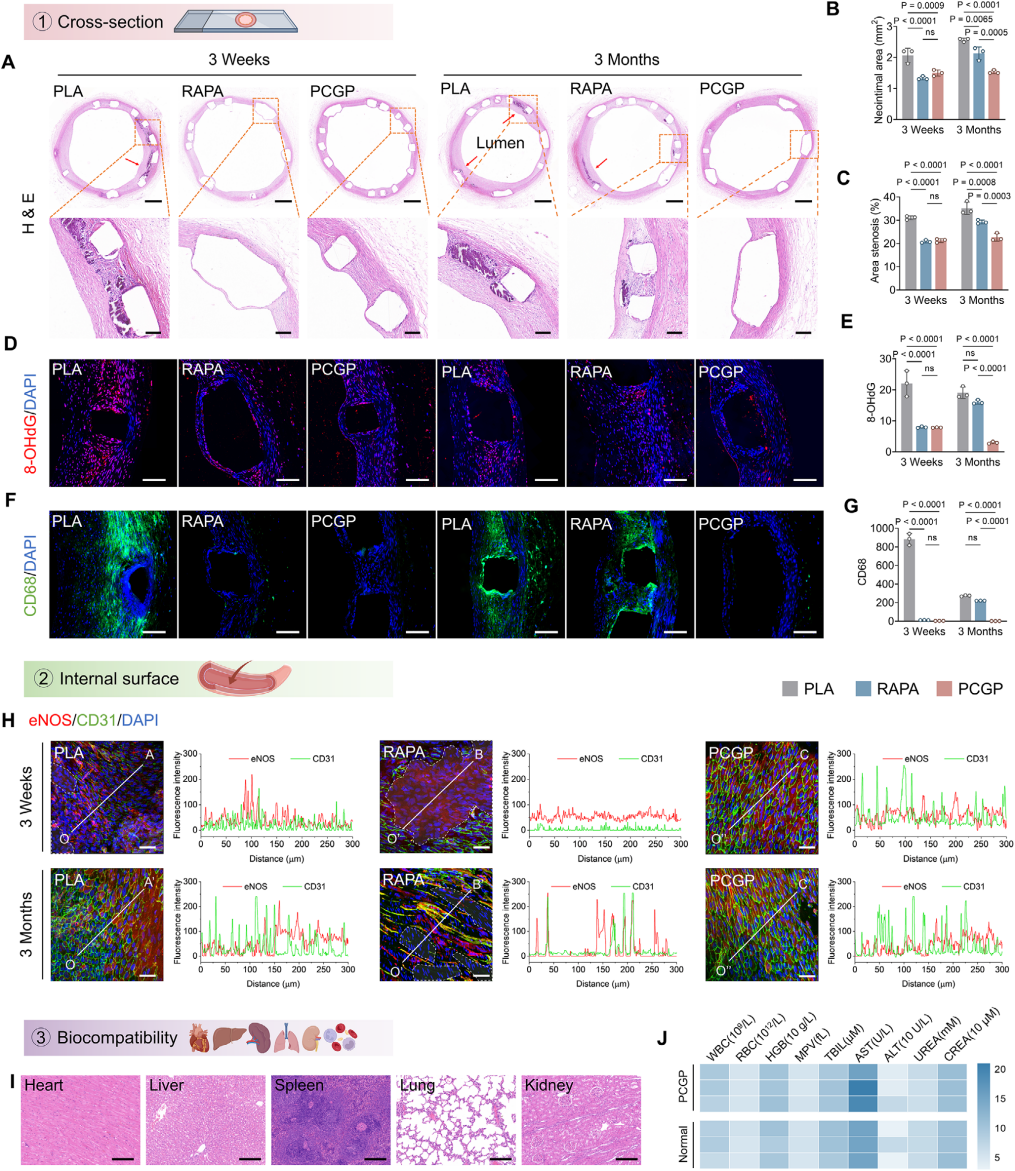
Biological effects of PCGP in rabbits. (A) H&E staining of stented vessels displaying neointimal formation. Scale bars, 500 µm (upper), 100 µm (lower). (B,C) Quantitative analysis of neointimal area and stenosis ratio from cross‐sectional images. (D,E) Immunofluorescence staining and quantitative assessment of oxidative stress marker 8‐OHdG showing reduced oxidative damage in stented abdominal aorta. Scale bar, 100 µm. (F,G) Immunofluorescence staining and quantitative assessment of CD68^+^ macrophage infiltration. Scale bar, 100 µm. (H) Confocal laser scanning microscopy (CLSM) of re‐endothelialization on the stent luminal surfaces. The fluorescence intensities of different cell components along the line segments (OA, O'B, O″C, OA′, O'B′ and O″C′) in the images are shown (red: eNOS, green: CD31). The dotted lines indicate the areas lacking continuous endothelial coverage. Scale bars, 50 µm. (I) H&E staining of major organs after 3 months of PCGP stent implantation showing no pathological abnormalities. Scale bar, 100 µm. (J) Hematological and biochemical analyses after 3 months of PCGP stent implantation confirming normal systemic parameters. Data are presented as mean ± standard deviation (*n*  =  3). Statistical significance was assessed by two‐way ANOVA with Šidák's multiple comparisons test. ns, no significance.

To probe oxidative stress and inflammatory responses, we analyzed oxidative stress biomarkers 8‐OHdG and macrophage marker CD68. At 3 weeks, oxidative stress and inflammation were prominent in bare stents, mitigated in PCGPs, and transiently suppressed in RAPAs (Figure 7D–G). At 3 months, oxidative damage and macrophage infiltration resurged around RAPAs but were markedly reduced in PCGPs, confirming durable antioxidant and anti‐inflammatory effects. The phenotypic stability of smooth muscle cells (SMCs) was assessed by α‐smooth muscle actin (α‐SMA) staining. PCGPs preserved high α‐SMA expression around stent struts, indicating preservation of the contractile phenotype and prevention of synthetic‐state remodeling (Figure S22). This stabilization mitigated matrix overproduction and vascular fibrosis, key drivers of restenosis.

Endothelial restoration was evaluated by confocal microscopy for CD31 (junctional marker) and eNOS (vasoregulatory enzyme). After 3 weeks, PCGPs supported complete endothelial coverage with elongated, aligned morphologies, whereas bare stents showed partial recovery and RAPAs exhibited pronounced delay (Figure 7H). Even at 3 months, RAPAs remained incompletely reendothelialized, a known risk factor for late thrombosis. PCGPs achieved near‐native endothelial organization and function, indicating preserved endothelial integrity by remodeling mitochondrial homeostasis (Figure S23 and Figure 1E).

Toxicological assessments confirmed systemic safety. Histopathology of major organs (heart, liver, spleen, lung, and kidney) revealed no pathological abnormalities (Figure 7I), and hematological and biochemical parameters, including white blood cell, red blood cell, platelet counts, and hepatic and renal function indices, remained within normal ranges (Figure 7J).

Taken together, these results delineate distinct healing trajectories: Bare stents elicited persistent inflammation and SMC dedifferentiation, driving neointimal hyperplasia. RAPAs transiently suppressed cell proliferation but impaired endothelial regeneration, leading to delayed late‐stage inflammation and restenosis. PCGPs, in contrast, maintained redox and immune homeostasis, preserved the contractile SMC phenotype, and promoted functional endothelial recovery, indicating that PCGP treatment shifted the injured vascular tissue toward a state more consistent with physiological vascular homeostasis. Collectively, these findings position the PCGP nanoshield as a promising biointerface for long‐term vascular healing.

## 3 Discussion

The durability of cardiovascular implants is fundamentally constrained by defective endothelial regeneration and persistent endothelial dysfunction. Although numerous surface modification strategies have aimed to enhance re‐endothelialization, the contribution of mitochondrial homeostasis to vascular repair has remained insufficiently defined. Here, our findings identify restoration of endothelial mitochondrial integrity as an important endothelial‐intrinsic axis associated with recovery following implantation.

PCGP was designed as a bioactive vascular interface capable of protecting endothelial mitochondria from implantation‐associated oxidative stress and thereby preserving the cellular programs required for sustained functional re‐endothelialization. Under oxidative stress, this intervention limits mitochondrial ROS accumulation, preserves membrane potential, and sustains respiratory function, thereby restoring cytoskeletal organization, intercellular junction integrity, and angiogenic competence [43]. In rabbit arterial stent models, PCGP reactivated the PI3K/AKT/eNOS‐NO axis, reinforcing endothelial barrier function and vascular homeostasis. Importantly, mitochondrial restoration was accompanied by coordinated modulation of thrombosis and inflammation. Citrate‐mediated calcium chelation attenuated thrombin activation and platelet aggregation, while polyphenolic components promoted macrophage polarization toward a reparative phenotype. These multilayered effects converged in vivo, resulting in functional re‐endothelialization and marked reduction of neointimal hyperplasia.

Several limitations should be acknowledged. Although the H_2_O_2_ model provided a controlled platform for studying endothelial oxidative injury, it did not fully recapitulate the complex vascular microenvironment following stent implantation, including chronic hemodynamic, thrombotic, inflammatory, and multicellular interactions. In addition, H_2_O_2_ induces broad oxidative stress rather than mitochondria‐specific injury. Although the rotenone model supports a functional role for mitochondrial dysfunction, future studies employing targeted genetic or pharmacological manipulation of mitochondrial quality‐control pathways and more physiologically relevant vascular models will further define the mechanistic basis and translational potential of this strategy. Moreover, integrative approaches such as transcriptomics and proteomics, particularly phosphoproteomics and redox proteomics, would help distinguish mitochondrial metabolic effects from other redox signaling mechanisms.

The use of HUVECs represents an additional limitation because venous and arterial endothelial cells differ in metabolism, mechanosensing, and responses to pulsatile shear stress. The effects of PCGP should therefore be further examined in primary arterial endothelial cells and under physiologically relevant flow conditions. In addition, p‐eNOS (Ser1177) alone does not provide a definitive measure of NO bioavailability or eNOS coupling. Future analyses incorporating eNOS Thr495 phosphorylation, eNOS dimer‐to‐monomer ratios, and direct NO detection would strengthen the mechanistic interpretation of the PI3K/Akt/eNOS–NO axis. Stable‐isotope tracing and targeted TCA‐cycle metabolomics will also be required to determine the cellular uptake, metabolic fate, and quantitative flux contribution of citrate released from PCGP.

The molecular mechanisms underlying the antithrombotic, immunoregulatory, and SMC‐modulatory effects of PCGP also remain incompletely defined. In this regard, investigating NADPH oxidase‐derived ROS could provide important mechanistic insights. PCGP shifted vascular healing toward a more physiological state, although direct transcriptomic comparison with uninjured vessels would further strengthen this conclusion, and direct in vivo comparison between PCG‐ and PCGP‐coated stents will be required to resolve their component‐specific contributions to endothelialization, inflammation, and neointimal remodeling.

Finally, the rabbit abdominal aorta model does not fully reproduce the pathological milieu of human coronary artery disease or restenosis. Future studies in hyperlipidemic, diabetic, and atherosclerotic animals, together with coronary implantation models and longer‐term follow‐up, will be required to establish the durability, mechanism, and clinical relevance of PCGP‐coated stents.

Collectively, our findings support restoration of endothelial mitochondrial homeostasis as an important component of PCGP‐mediated vascular repair and provide a framework for integrating endothelial metabolic recovery with complementary regulation of thrombosis, inflammation, and vascular remodeling.

## 4 Conclusion

In summary, by integrating single‐cell RNA‐sequencing data from rat carotid balloon injury with ultrastructural and histological evidence from rabbit stented arteries, we identified mitochondrial disorganization and dysfunction as common features of injured endothelial cells. Guided by these findings, we developed a bioactive poly(citrate‐polyglycol‐procyanidin) nanoshield that integrates endothelial mitochondrial protection with antithrombotic and immunoregulatory functions for cardiovascular implants. PCGP attenuated oxidative injury, preserved mitochondrial ultrastructure and membrane potential, restored mitochondrial respiration and glycolytic activity, and supported endothelial survival and angiogenic capacity. In rabbit arterial stent models, PCGP‐coated stents promoted endothelial coverage with restored CD31 and eNOS expression, and activated the PI3K/AKT/eNOS–NO signaling axis. Collectively, these findings support restoration of endothelial mitochondrial homeostasis as an important mechanistic axis associated with vascular repair and establish PCGP as a multifunctional regenerative interface for cardiovascular devices.

## 5 Methods

### 5.1 Single‐Cell RNA Sequencing (scRNA‐seq) Data Analysis

Publicly available single‐cell RNA‐sequencing data from rat carotid arteries following balloon injury were obtained from the Gene Expression Omnibus (GEO) database (GSE174098). The processed 10x Genomics expression matrix was analyzed using Seurat v5.5.1. After quality control, data were normalized using the LogNormalize method with a scale factor of 10,000. Clusters were categorized into key cell types based on the expression of canonical markers. DEG analyses, GO and KEGG pathway enrichment analyses, and GSEA were further performed in endothelial cells. Gene sets associated with mitochondrial homeostasis and endothelial injury responses were curated from relevant GO terms. Single‐cell gene‐set scores were calculated using the AddModuleScore function in Seurat, and differences between experimental groups were assessed using the Wilcoxon rank‐sum test. Because only one pooled sequencing library was available for each condition, the single‐cell analysis was treated as exploratory and descriptive.

### 5.2 Materials

Citric acid (CA) was obtained from Sigma‐Aldrich. 3‐Hydroxytyramine hydrochloride (DA), 1,6‐hexanediamine (HD), 1,8‐octanediol (OD) and procyanidin (PA) were purchased from Shanghai Titan Scientific Co., Ltd. Polyethylene glycol 200 (PEG), 1‐ethyl‐3(3‐dimethyl aminopropyl)carbodiimide (EDC), and N‐hydroxysuccinimide (NHS) were purchased from Shanghai Aladdin Biochemical Technology Co., Ltd. Poly(l‐lactic acid) (PLA) stents were provided by Sichuan XingTai Pule Medical Equipment Co., Ltd. Ultrapure (18.25 MΩ cm) water was used in all experiments.

### 5.3 Preparation of PCGP

Poly(citrate‐polyglycol‐procyanidin) (PCGP) was synthesized through melt‐derived polymerization using CA, OD, PA, and PEG in a molar ratio of CA: OD: PA: PEG = 1: 0.2: 0.06: 1. Poly(citrate‐polyglycol) (PCG) was synthesized using CA, OD, and PEG in the same molar ratios. Briefly, CA, OD, PA, and PEG were melted at 160°C for 20 min with a constant flow of nitrogen gas and reacted at 140°C for another 7 h under vacuum with stirring. The resulting polymer was purified by dialysis for 3 days using a dialysis tube (MWCO 1000 Da) after centrifugation at 1500 rpm for 10 min.

Before fabricating the PCGP nanoshield, substrates were ultrasonically cleaned with acetone and ultrapure water, then immersed in dopamine solution (2 mg/ml DA and 1 mg/ml HD in Tris buffer solution of pH 8.5) for 12 h, followed by cleaning with ultrapure water. The as‐prepared coating, rich in amino groups, was named PDA. PCGP polymer was grafted onto the PDA‐modified substrates via carbodiimide chemistry and electrostatic interactions. Briefly, PCGP (1 mM) was added to a solution containing EDC (10 mM) and NHS (4 mM), and the PDA‐treated samples were immersed in this solution for 12 h. The samples were washed with PBS for subsequent experiments, resulting in the PCGP nanoshield. PCG‐coated surfaces were prepared using the same procedure.

### 5.4 Characterization

The chemical structure of the PCGP polymer was characterized using ^1^H NMR (Ascend 600 M, Bruker) and ATR‐FTIR (Spectrum TWO, PerkinElmer). The molecular weight was measured by gel permeation chromatography (1260 Infinity II, Agilent) in the tetrahydrofuran phase.

The real‐time assembly process of the PCGP nanoshield was monitored using QCM‐D (Qsense Analyzer, Biolin). Surface morphology was examined by SEM (Apreo S HiVac, Thermo Fisher Scientific), and surface chemical composition was analyzed by XPS (ESCALAB Xi+, Thermo Fisher Scientific). The micromorphology and roughness of the coating were verified by AFM (Dimension ICON, Bruker). Hydrophilicity was assessed using a contact angle meter (Theta, Biolin) and surface free energy (γ) was calculated according to Owens' two‐liquid method, based on contact angle measurements with water and diiodomethane. The coating thickness on silicon wafers was measured using an ellipsometer (UVISEL PLUS, Horiba). The concentration of citric acid released from the coating was detected using an Amplex Red Citrate Assay Kit (Beyotime). Detailed experimental procedures are provided in the Supporting Information.

### 5.5 Free Radical‐Scavenging Assays

For free radical‐scavenging assays, DPPH (0.1 mM) was dissolved in anhydrous ethanol. Bare and coated samples were incubated with 500 µL of DPPH solution for 1 h at 37°C, away from light. After incubation, 200 µL of the solution was collected, and the absorbance at 517 nm was measured. The DPPH radical scavenging ability was calculated using the formula:

$$\%\mathrm{DPPH}\;\mathrm{radical}\;\mathrm{scavenging}\;\mathrm{activity}=\frac{A_{C}-A_{S}}{A_{C}}\times 100\%$$

where A_C_ and A_S_ are the absorbance values of the control and sample, respectively.

### 5.6 Cells and Animals

Human umbilical vein endothelial cells (HUVECs) were purchased from Shanghai Zhongqiao Xinzhou Biotechnology Co., Ltd. Mouse mononuclear macrophage cells (RAW 264.7) were purchased from Cloud‐clone Technology Co., Ltd. Male adult New Zealand white rabbits (2.5–3 kg, ∼ 3 months old) and male Sprague Dawley rats (SD rats, ∼ 250–300 g, ∼ 10 weeks old) were supplied by Chengdu Dossy Experimental Animals co., Ltd. All animal experiments were conducted in accordance with strict protocols approved by the Medical Ethics Committee of Sichuan Province, and complied with the guidelines for the care and use of laboratory animals set by Sichuan University (No. KS20240287).

### 5.7 Analysis of ROS Scavenging

The PCG and PCGP coatings were prepared on the cell culture plates or confocal discs. After 12 h of ultraviolet sterilization, a HUVEC suspension at a density of 2 × 10^4^ cells/mL was seeded onto different surfaces. To simulate the effect of ROS on endothelial cell growth after cardiovascular device implantation, 400 µM H_2_O_2_ was added to the medium after 48 h of culture, and cells were further cultured for 6 h. DCFH‐DA (MedChemExpress), DHE (MedChemExpress) and MitoSOX (MedChemExpress) probes were used to measure ROS levels in HUVECs and mitochondria, respectively. Briefly, HUVECs from different groups were incubated with these probes in the dark at 37°C for 30 min, then stained with Hoechst 33342 (Beyotime) for 5 min. ROS levels were detected using an inverted fluorescence microscope (IX73, Olympus) or CLSM (ZEISS LSM880, Carl Zeiss).

### 5.8 Measurement of MMP

After 6 h of stimulation with 400 µM H_2_O_2_, HUVECs on different sterilized surfaces were incubated with a JC‐1 dye (MedChemExpress) working solution at 37°C for 20 min. After washing with PBS, cells were observed under a confocal microscope for the red fluorescence of J‐aggregates and green fluorescence of J‐monomer, separately. Semiquantitative analysis and MMP calculations (red/green fluorescence intensity ratio) were performed using ImageJ software.

### 5.9 qRT‐PCR Array

Total RNA was extracted from HUVECs or RAW 264.7 using the RNA extraction kit (Yeasen) and quantified using the NanoDrop 2000 (Thermo Fisher Scientific). RNA was reverse‐transcribed into cDNA using cDNA Synthesis SuperMix (Yeasen). The synthesized cDNA was then combined with specific primers and SYBR Green Master Mix (Yeasen). qRT‐PCR was performed using the QuantStudio 3 system (Thermo Fisher Scientific), and relative gene expression was quantified using the 2^−ΔΔCt^ method.

To assess mitochondrial function recovery and inflammation in HUVECs, the expression of genes such as *MDH2*, *ACO2*, *OGDH*, *ND1*, *NDUFA4*, *SDHB*, *SOD2*, *PRDX3*, *NQO1*, *IL1B*, and *IL6* were quantified after 6 h of H_2_O_2_ (400 µM) stimulation. To characterize macrophage phenotypes, we quantified proinflammatory factors (*TNF‐α* and *iNOS*) and anti‐inflammatory factors (*CD206* and *IL‐4*) after 12 h of LPS (1 µg/mL, Sigma–Aldrich) stimulation. Primers used for qRT‐PCR analysis in HUVECs are listed in Table S2. Primers related to macrophage polarization are listed in Table S3.

### 5.10 TEM

Cell samples or vessel samples were pre‐fixed with 2.5% glutaraldehyde and post‐fixed with 1% osmium tetroxide. After dehydration with acetone, the samples were embedded in Epon‐812 medium. Ultrathin sections were cut using a Leica UC7 ultramicrotome, and stained with uranyl acetate for 15 min and lead citrate for 2 min at room temperature. TEM images were acquired using a JEM‐1400FLASH transmission electron microscope (JEOL, Japan).

### 5.11 Cellular Apoptosis Detection

HUVECs were cultured on different surfaces for 48 h, followed by the addition of 400 µM H_2_O_2_ for an additional 6 h. Adherent cells were observed with a fluorescence microscope after staining with FDA and PI. Cell viability was evaluated using a CCK‐8 assay and apoptotic cells were detected using an Annexin V‐FITC/PI apoptosis detection kit (Yeasen) and quantified by flow cytometry (BD FACSCelesta, BD Biosciences).

### 5.12 Seahorse Analysis

Sterilized PCG and PCGP‐coated culture plates were soaked in the medium at 37°C for 1 day to prepare extracts. A HUVEC suspension and extracts (v/v, 1/1) were added in Seahorse XFe96 cell culture microplates and cultured for 24 h. After 6 h of stimulation with 400 µM H_2_O_2_, the Seahorse XFe96 Flux Analyzer (Seahorse Bioscience, Agilent, USA) was used to assess the OCR and ECAR of HUVECs following the manufacturer's protocol. Before OCR experiment, the culture medium was replaced with Seahorse XF assay medium supplemented with pyruvate (1 mM), glutamine (2 mM), and glucose (10 mM). Cells were equilibrated in a non‐CO_2_ incubator at 37°C for 45–60 min before measurement. Subsequently, the real‐time OCR was conducted through sequential injection of several ETC modulators: oligomycin (1 µM), carbonyl cyanide p‐trifluoromethoxyphenylhydrazone (0.5 µM), rotenone (0.5 µM), and antimycin A (0.5 µM). For glycolytic rate measurement, the culture medium was replaced with Seahorse XF assay medium, and proton efflux rate (PER) and glycolytic proton efflux rate (glycoPER) were determined using the Seahorse XF Glycolytic Rate Assay. Rotenone/antimycin A and 2‐deoxy‐D‐glucose were sequentially injected to calculate basal glycolysis, compensatory glycolysis, and post‐2‐DG acidification. OCR and ECAR values were normalized to cell number.

### 5.13 Cellular Immunofluorescence Staining

Cells grown on different surfaces were fixed with 4% paraformaldehyde for 15 min. After washing with PBS, cells were blocked with 5% normal goat serum for 1 h. Subsequently, primary antibodies were applied overnight at 4°C, followed by incubation with secondary antibodies for 2 h at room temperature. Cells were stained with DAPI for 5 min. Primary antibodies used include anti‐VE‐cadherin (83766‐2‐RR, Proteintech), anti‐CD86 (DF6332, Affinity) and anti‐CD206 (DF4149, Affinity).

### 5.14 Tube Formation

After 6 h of stimulation with 400 µM H2O2, HUVECs on different sterilized surfaces were harvested. Matrigel (Corning) was spread on the bottom of the pre‐cooled cell culture plate and then placed at 37°C for 1 h to solidify. A HUVEC suspension was added to the Matrigel surface and incubated for 8 h. After FDA staining, tube formation was observed using a CLSM, and the number of junctions and total tube lengths were analyzed using ImageJ software.

### 5.15 Migration Assay

A HUVEC suspension at a density of 2 × 10^5^ cells/mL was seeded onto different surfaces and cultured until the intact cell layer was formed. Scratch wounds were created using a 1 mL sterile pipette tip, and 400 µM H_2_O_2_ was then added. After 6 h, H_2_O_2_ was removed and replaced with fresh low serum culture medium. After 48 h of cultivation, the scratch size was measured to assess cell migration for each condition utilizing an inverted fluorescence microscope after FDA staining.

### 5.16 Flow Cytometric Detection of E‐selectin

HUVECs from each group were collected, washed with PBS, and resuspended in 100 µL PBS. Cells were incubated with FITC‐conjugated anti‐E‐selectin (FITC‐98189, Proteintech) at 4°C for 30 min in the dark. For each sample, 5 µL of the antibody was added. After washing and resuspension in PBS, the expression of E‐selectin was analyzed using flow cytometry, and data were processed with FlowJo software to determine the percentage of positive cells.

### 5.17 Antithrombogenicity Test

For in vitro evaluation, the bare, PCG, and PCGP‐coated stainless‐steel sheets or PVC tubes were incubated with BSA‐FITC (1 mg/mL; Solarbio) at 37°C for 2 h in the dark. After rinsing with PBS, the proteins adsorbed onto the surfaces were quantified using a microplate reader (Synergy H1, BioTek), exciting at 488 nm and emitting at 525 nm, and qualitatively observed using a fluorescence microscope.

Whole blood was obtained from a healthy adult New Zealand Rabbit. Platelet‐rich plasma (PRP) was collected by centrifuging fresh rabbit blood at 1500 rpm for 15 min. The samples were incubated with PRP or whole blood at 37°C for 1 h, followed by gentle rinsing with PBS. The adhesion and morphology of platelets and erythrocytes on the coatings were observed by SEM. To test the coating's ability to chelate calcium ions, the materials were immersed in 2.6 mmol/L CaCl_2_ and rinsed with ultrapure water. Finally, calcium content on the surface was determined by XPS analysis.

For *ex vivo* evaluation, the coatings were prepared on 316LSS foils (10 mm × 8 mm × 0.5 mm) and placed inside a PVC catheter, ensuring full contact between the sample's backside and the inner catheter wall. New Zealand white rabbits were anesthetized by intravenous administration of pentobarbital sodium (2 mL, 3%) via the ear vein. The left carotid artery and right external jugular vein were surgically exposed, and the PVC catheter was connected to the artery and vein through the indwelling needle to form a circulation loop. After 1 h of continuous blood circulation, the samples were removed, gently rinsed with normal saline, and subsequently analyzed using SEM to evaluate thrombus formation.

### 5.18 Subcutaneous Implantation Test

Bare and PCGP‐coated PLA sheets were implanted subcutaneously into the dorsal region of SD rats. After 2 weeks, the implanted samples with the surrounding fibrous tissues were harvested and fixed with 4% paraformaldehyde. The fibrotic tissues in direct contact with the implanted samples were embedded in paraffin, sectioned, and subjected to histological analysis via H&E staining, along with immunofluorescence staining using anti‐CD3 (GB11014, Servicebio), anti‐CD68 (GB113109, Servicebio), anti‐IL‐10 (GB11108, Servicebio) and anti‐TNF‐α (GB11188, Servicebio). Image acquisition, fields selection, and quantitative assessment were performed by investigators blinded to treatment allocation.

### 5.19 Vascular Stent Deployment in Rabbits

Rabbits were randomly divided into three treatment groups at each time (*n* = 3 per group): PCGP‐coated PLA stents, Rapamycin (RAPA)‐coated PLA stents, and bare PLA stents as controls. RAPA‐coated stents were prepared by loading rapamycin (5 mg/mL) directly onto bare PLA stents using an ultrasonic spraying system, according to our previously reported procedure [44]. The rapamycin loading density was 58.27 µg/cm^2^. All stents were sterilized by irradiation before implantation. General anesthesia was induced by injection of pentobarbital sodium through the marginal ear vein (25 mg/mL, 0.8 mL/kg). An approximately 3 cm segment of the femoral artery was surgically exposed, and a balloon catheter‐mounted stent was introduced through the femoral artery and advanced into the abdominal aorta. The stent was deployed by balloon inflation at 10 atm for 40 s. The catheter was subsequently withdrawn, and the femoral artery was ligated. After surgery, ampicillin sodium (10 mg/mL, 1 mL/kg) was administered intramuscularly. Aspirin (10 mg/kg) and clopidogrel (3 mg/kg) were administered once daily for three consecutive days after implantation. At 3 weeks and 3 months after implantation, the rabbits were euthanized with an overdose of pentobarbital sodium, and the stented abdominal aortic segments were collected for subsequent analyses. The explanted stents and surrounding vascular tissues were subjected to histological analysis through H&E staining, as well as immunofluorescence staining with anti‐8‐OHdG (sc‐66036, Santa Cruz Biotechnology), anti‐α‐SMA antibody (BM0002, Boster Bio), anti‐CD68 antibody (916104, BioLegend), anti‐CD31 antibody (ab9498, Abcam), and anti‐eNOS (ab5589, Abcam). Corresponding statistics were calculated using ImageJ software, with the healthy rabbit's abdominal aorta as the reference (set to 1.0). Image acquisition, fields selection, and quantitative assessment were performed by investigators blinded to treatment allocation.

### 5.20 RNA‐Sequencing and Data Analysis

After 3 weeks of implantation, vascular tissues surrounding the stents (*n* = 3 per group) were harvested and immediately snap‐frozen in liquid nitrogen. Library construction and sequencing procedure were performed by Guangzhou Epibiotek Co., Ltd. Differential gene expression analysis was conducted using the DESeq2 R package. GO and KEGG analyses were carried out using the clusterProfiler R package (v3.6.0).

### 5.21 Western Blot

To verify whether PCGP promoted endothelialization through the PI3K/AKT/eNOS signaling pathway, HUVECs were divided into four groups: Control, H_2_O_2_, H_2_O_2_ + PCGP, and H_2_O_2_+LY+PCGP. Cells in the H_2_O_2_+PCGP and H_2_O_2_+LY+PCGP groups were cultured on PCGP‐coated surfaces. For pathway inhibition, cells in the H_2_O_2_+LY+PCGP group were pretreated with the PI3K inhibitor LY294002 (10 µM, MedChemExpress) for 1 h before oxidative stimulation. Except for the Control group, all groups were exposed to 400 µM H_2_O_2_ for 6 h. Total cellular proteins were extracted using RIPA lysis buffer (Beyotime) containing protease and phosphatase inhibitors. Protein concentration was determined using the BCA protein assay kit (Beyotime). Equal amounts of protein were separated on a 10% SDS‐PAGE gel and transferred to PVDF or nitrocellulose membranes. After blocking with 5% non‐fat milk for 1 h, the membrane was incubated with the primary antibody at 4°C overnight. The membrane was then incubated with the secondary antibody for 1 h. Protein bands were visualized using an enhanced chemiluminescence reagent kit (Beyotime). Primary antibodies used were as follows: PI3K p85 (4292, Cell Signaling Technology), phospho‐PI3K p85 (HA721672, HUABIO), AKT (10176‐2‐AP, Proteintech), phospho‐AKT (Ser473; 66444‐1‐Ig, Proteintech), eNOS (27120‐1‐AP, Proteintech), and phospho‐eNOS (Ser1177; HA721188, HUABIO).

### 5.22 NO Assay

NO levels in HUVEC culture supernatants were determined using a Colorimetric NO Assay Kit (APExBIO). Briefly, cell culture supernatants were collected, and any debris was removed by centrifugation. Sodium nitrite standards were prepared for standard curve generation. Samples or standards (150 µL) were mixed with sterile water (130 µL) and freshly prepared Griess reagent (20 µL; Griess Reagent A:B = 1:1). After incubation at room temperature for 30 min in the dark, the absorbance was measured at 540 nm using a microplate reader. NO concentrations were calculated according to the standard curve.

### 5.23 Statistical Analysis

All data are presented as mean ± SD. Statistical differences were analyzed using GraphPad Prism 10.1. Unpaired two‐tailed Student's t‐test was used for comparisons between two groups. One‐way ANOVA with Tukey's multiple comparisons was used when comparing three or more groups within one factor. Two‐way ANOVA with Šidák's multiple‐comparisons test was used for experiments involving two independent factors. The probability value was represented by P, and P values < 0.05 were considered statistically significant. The statistical methods used in each experiment and the corresponding statistical analysis results were listed in the figure legends.

## Author Contributions

**Xiyu Liu**: conceptualization, methodology, software, data curation, investigation, validation, formal analysis, visualization, writing – original draft. **Wen Zhang**: conceptualization, methodology, software, validation, visualization, formal analysis. **Lunxin Liu**: supervision, project administration. **Lan Gui**: investigation, methodology, software, formal analysis, validation. **Yumei Qin**: methodology, software, validation, formal analysis. **Xiaohui Yang**: methodology, software, formal analysis, validation, visualization. **Longjian Zhang**: validation, software, data curation. **Chaohua Wang**: project administration, supervision. **Jinpeng Hu**: supervision, project administration. **Gaoyang Guo**: supervision, project administration. **Qing Jiang**: project administration, supervision. **Jin Wang**: project administration, supervision. **Li Yang**: project administration, supervision. **Bo Zhang**: funding acquisition, resources, project administration, writing – review and editing, supervision, conceptualization. **Yunbing Wang**: funding acquisition, resources, project administration, supervision.

## Conflicts of Interest

The authors declare no conflicts of interest.

## Supporting information




**Supporting File**: advs77638‐sup‐0001‐SuppMat.docx.

## Data Availability

The data that support the findings of this study are available from the corresponding author upon reasonable request.
