# Supplementary material for: Restoring Endothelial Mitochondrial Homeostasis With a Bioactive Nanoshield Promotes Vascular Regeneration on Cardiovascular Implants
Source: Adv Sci (Weinh). 2026 Oct 25:e77638. Online ahead of print. doi: 10.1002/advs.77638 (PMC13696785; doi:10.1002/advs.77638)
Supplement: Supplementary file 1 [file ADVS-9999-e77638-s001.docx]

**Supporting Information**

**Restoring Endothelial Mitochondrial Homeostasis with a Bioactive Nanoshield Promotes Vascular Regeneration on Cardiovascular Implants**

*Xiyu Liu^1^, Wen Zhang^1^, Lunxin Liu^2^, Lan Gui^1^, Yumei Qin^1^, Xiaohui Yang^1^, Longjian Zhang^1^, Chaohua Wang^2^, Jinpeng Hu^1^, Gaoyang Guo^1^, Qing Jiang^1^, Jin Wang^3^, Li Yang^1^, Bo Zhang^1^* and Yunbing Wang^1^**

^1^ National Engineering Research Center for Biomaterials, Sichuan University, Chengdu, Sichuan 610065, China

^2^ Department of Neurosurgery, West China Hospital of Sichuan University, Chengdu, Sichuan 610044, China

^3^ Key Lab of Advanced Technology of Materials of Education Ministry, Southwest Jiaotong University, Chengdu, Sichuan 610031, China

* Corresponding author: bo[zhang@scu.edu.cn](mailto:zhang@scu.edu.cn); yunbing.wang@scu.edu.cn

# Supplementary methods

## M1. QCM-D measurement

The quartz crystal microbalance with dissipation (QCM-D) was used to monitor the adsorption and desorption behaviors of coating components on the surfaces of AT-cut 5 MHz gold-plated quartz crystals (Φ 10 mm) in situ in real time. Briefly, the PDA-modified quartz crystals were loaded into the flow modules and ultrapure water was continuously injected into the flow modules at a constant flow rate of 50 μL/min by the peristaltic pump at 25°C until the frequency variation (ΔF) was less than 1 Hz within two minutes. Then a freshly prepared PCGP solution (1 mM PCGP, containing 10 mM EDC and 4 mM NHS) was injected into the chambers at the same flow rate and temperature until adsorption equilibrium. Subsequently, ultrapure water was injected to remove loosely adsorbed molecules. After the experiment, the quartz crystals were removed, and the channels were cleaned thoroughly with 2% sodium dodecyl sulfate (SDS) solution and ultrapure water in sequence and emptied. Data analysis was conducted with Dfind Smartfit modeling in QSense Dfind software.

## M2. Surface free energy test

Surface free energy (γ) was calculated according to Owens two-liquid method by measuring the contact angles of water and diiodomethane. The calculations were performed using the following equations:

$\boldsymbol{\gamma}_{\boldsymbol{s}}\boldsymbol{=}\boldsymbol{\gamma}_{\boldsymbol{s}}^{\boldsymbol{p}}\boldsymbol{+}\boldsymbol{\gamma}_{\boldsymbol{s}}^{\boldsymbol{d}}$	(1-1)

$\boldsymbol{\gamma}_{\boldsymbol{L}}\left( \boldsymbol{1+cos\theta} \right)\boldsymbol{=2}\sqrt{\boldsymbol{\gamma}_{\boldsymbol{s}}^{\boldsymbol{p}}\boldsymbol{\gamma}_{\boldsymbol{L}}^{\boldsymbol{p}}}\boldsymbol{+2}\sqrt{\boldsymbol{\gamma}_{\boldsymbol{s}}^{\boldsymbol{d}}\boldsymbol{\gamma}_{\boldsymbol{L}}^{\boldsymbol{d}}}$	(1-2)

where θ is the static contact angle, and L and S represent liquid and solid phases, respectively. γ_L_ (mJ/m^2^), γ_L_^p^ (mJ/m^2^), and γ_L_^d^ (mJ/m^2^) are the total, polar, and dispersive surface free energy of water and diiodomethane. γ_s_ (mJ/m^2^), γ_s_^P^ (mJ/m^2^), and γ_s_^d^ (mJ/m^2^) are the total, polar, and dispersive surface free energy of the coated surface.

## M3. Stability test

To evaluate the stability of the PCGP coating under continuous dynamic circulation, a peristaltic pump-driven circulation system was constructed to mimic the continuous shear stress exerted by blood flow. In brief, PDA- and PCGP-coated 316LSS foils (10 mm × 8 mm × 0.5 mm) were placed inside commercially available polyvinyl chloride catheters, with the back of the sheet tightly attached to the inner wall of the catheter. The catheter was connected to a peristaltic pump and PBS reservoir to establish a closed-loop circulation system. After circulation for 0, 7, 14, and 28 days, the samples were retrieved, rinsed, dried, and subjected to XPS (ESCALAB Xi+, Thermo Fisher Scientific).

## M4. Measurement of intracellular citrate

PCG and PCGP coatings were prepared on cell culture plates. After ultraviolet sterilization for 12 h, HUVECs were seeded onto the different surfaces at a density of 2 × 10⁴ cells mL⁻¹. After 48 h of culture, the cells were exposed to 400 μM H₂O₂ for an additional 6 h. Intracellular citrate concentrations were measured using an Amplex Red Citrate Assay Kit (Beyotime, China) according to the manufacturer’s instructions.

## M5. Rotenone injury model

To establish a mitochondria-specific injury model, HUVEC viability was assessed after exposure to increasing concentrations of rotenone for 24 h using the CCK-8 assay. Based on the dose-response results, 500 nM rotenone was selected for subsequent experiments. PCG and PCGP coatings were prepared on cell culture plates or confocal discs and ultraviolet sterilized for 12 h. HUVECs were assigned to the Control, rotenone (R), R+PCG, and R+PCGP groups. Cell viability was determined by measuring absorbance at 450 nm. For the tube-formation assay, HUVECs were seeded onto Matrigel-coated plates, and capillary-like networks were imaged and quantified for junction number and total tube length using ImageJ. Endothelial junction integrity was evaluated by immunofluorescence staining for VE-cadherin, and fluorescence intensity was quantified using ImageJ.

## M6. Photon-counting CT

*In vivo* imaging of New Zealand white rabbits was performed using the Naeotom Alpha Photon-counting CT. Rabbits were anesthetized with pentobarbital, and iodinated non-ionic CT contrast (Iomeprol) was administered intravenously at a dose of 1 mL/kg body weight to enhance vascular visualization. After scanning, images were reconstructed into 3D models for analysis of vascular structures.

## M7. Measurement of inflammatory cytokines

The PCG and PCGP coatings were prepared on the cell culture plates. After 12 h of ultraviolet sterilization, RAW 264.7 macrophages were seeded on different surfaces. The levels of inflammatory cytokines in cell culture supernatants were measured using ELISA kits according to the manufacturers’ instructions. Briefly, RAW 264.7 macrophages were stimulated with LPS (1 μg/mL) for 12 h, and the culture supernatants were collected to determine TNF-α and IL-6 levels using ELISA kits (Jianglai Bio). The absorbance was measured using a microplate reader, and cytokine concentrations were calculated according to the standard curves.

# Supplementary figures

**Fig. S1. Quality control, cellular annotation, and endothelial functional enrichment analysis of GSE174098.**

(**A**) Violin plots showing the distributions of nFeature_RNA, nCount_RNA, and mitochondrial gene percentage before and after quality control in the control and injury groups.

(**B**) t-SNE visualization of single-cell transcriptomes from rat carotid arteries.

(**C**) Dot plot showing the expression of canonical cell-type markers across clusters.

(**D**) Feature plots showing the expression patterns of endothelial markers *Cdh5*, *Cldn5*, and *Sox17* on the t-SNE map.

(**E**) KEGG enrichment analysis of differentially expressed genes in endothelial cells between the injury and control groups.

(**F**) Gene set enrichment analysis showing injury-associated changes in endothelial cells.

**Fig. S2. ^1^H NMR spectrum of PCGP supporting the presence of characteristic methylene and aromatic peaks.**

**Fig. S3. ATR-FTIR analysis of PCG and PCGP, showing the additional phenyl-ring-associated band at 1520 cm⁻¹ in PCGP.**

**Fig. S4. AFM images and corresponding line profile showing smooth surface characteristics of unmodified substrates (n = 3).**

**Fig. S5. (A) Schematic illustration of the** **peristaltic pump-based circulation system. (B) Surface nitrogen atomic percentages of PDA- and PCGP-coated samples determined by XPS after circulation for 0, 7, 14, and 28 days.**

**Fig. S6. Cumulative citrate release from PCGP-coated samples over 28 days.**

**Fig. S7. SEM images of PCGP-coated stents displaying a continuous nanostructured surface. Scale bar, 100 μm.**

**Fig. S8. Schematic illustration of the *in vitro* evaluation of mitochondrial homeostasis in endothelial cells.**

**Fig. S9. Expression of antioxidant enzyme-related genes in HUVECs under oxidative stress.**

**Fig. S10. Intracellular citrate levels in HUVECs under oxidative stress.**

**Fig. S11. (A) Representative extracellular acidification rate (ECAR) profiles of HUVECs measured using Seahorse extracellular flux analysis. (B) Quantification of glycolytic parameters in HUVECs in different groups.**

**Fig. S12.** **(A) FDA/PI live/dead staining of HUVECs. (B) CCK-8 assessment of cell viability in the indicated groups.**

**Fig. S13. PCGP rescues endothelial viability, angiogenic capacity, and intercellular junction integrity under rotenone-induced mitochondrial dysfunction.**

(**A**) CCK-8 assessment of HUVEC viability following exposure to the indicated concentrations of rotenone and different treatments. Based on the dose-response results, 500 nM rotenone was selected to induce mitochondrial injury in the R group.

(**B**) Representative fluorescence images of endothelial tube formation and corresponding quantification of junction number and total tube length. Scale bar, 200 μm.

(**C**) Representative immunofluorescence staining of VE-cadherin and corresponding fluorescence-intensity quantification. Scale bar, 50 μm.

**Fig. S14. Uncropped Western blot images used in Figure 5H.**

**Fig. S15. Illustration of *in vitro* antithrombogenicity experiments.**

**Fig. S16. (A and B) SEM images and quantitative analysis of attached erythrocytes. Scale bar, 10 μm.**

**(C and D) SEM images and quantitative analysis of attached platelets. Scale bar, 10 μm.**

**Fig. S17. TRITC-conjugated phalloidin (red) and DAPI (blue) staining of macrophages cultured on samples. Scale bar, 20 μm.**

**Fig. S18. qRT-PCR analysis of mRNA expression levels of *Tnf*, *Nos2*, *Mrc1*, and *Il4* indicating a shift toward an anti-inflammatory M2 phenotype in the PCGP group.**

**Fig. S19. ELISA analysis of IL-6 and TNF-α protein levels showing modulation of cytokine secretion by PCGP.**

**Fig. S20. PCGP exhibits potent immunoregulatory effects by promoting macrophage M2 polarization and suppressing M1 activation.**

**Fig. S21. Representative photographs of explanted arteries at 3 months after stent implantation. Scale bar, 5 mm.**

**Fig. S22. Immunofluorescence staining and quantification of α-SMA expression indicating maintenance of the contractile SMC phenotype. Scale bar, 100 μm.**

**Fig. S23. Quantification of eNOS and CD31 expression indicating re-endothelialization across stent groups.**

# Supplementary tables

**Table S1.** The elemental content of coatings.

| Sample name | C (%) | N (%) | O (%) |
| --- | --- | --- | --- |
| PDA | 72.53 | 10.10 | 17.37 |
| PCG | 64.48 | 5.70 | 29.82 |
| PCGP | 65.05 | 2.01 | 32.94 |

**Table S2.** Primers related to mitochondrial function.

| Primer name | Species | Orientation | Primer sequence (5’-3’) |
| --- | --- | --- | --- |
| GAPDH | H | FORWARD | GGAAGCTTGTCATCAATGGAAATC |
|  |  | REVERSE | TGATGACCCTTTTGGCTCCC |
| SOD2 | H | FORWARD | AGGGAGATGTTACAGCCCAGAT |
|  |  | REVERSE | CAATTTGTAAGTGTCCCCGTTC |
| PRDX3 | H | FORWARD | TGAAGTTGTCGCAGTCTCAGT |
|  |  | REVERSE | GGACTTGGCTTGATCTTAGTGC |
| NQO1 | H | FORWARD | TGCCGAGTCTGTTCTGGCTTAT |
|  |  | REVERSE | ATGGCAGCGTAAGTGTAAGCAA |
| MDH2 | H | FORWARD | CGGGCAGCCACTTTCACTT |
|  |  | REVERSE | GGTCCGAGGTAGCCTTTCACA |
| ACO2 | H | FORWARD | CATCCGAGTGGGTCTAATTGGTA |
|  |  | REVERSE | GGAACCTGGAGTGATGGTGAAC |
| OGDH | H | FORWARD | ACAAGTCTAGTGAGAATGGCGTGG |
|  |  | REVERSE | CAGGTGGTACTTCACATCTCCG |
| ND1 | H | FORWARD | GCCTCCTATTTATTCTAGCCACCT |
|  |  | REVERSE | TGATAAGGGTGGAGAGGTTAAAGG |
| NDUFA4 | H | FORWARD | GTATCTCTTGCGTCTGGCATTGT |
|  |  | REVERSE | GCTTGCTGTAATCCACATTCACTG |
| SDHB | H | FORWARD | TCAGGAAGGCAAGCAGCAGTATC |
|  |  | REVERSE | GCTCCTCTGTGAAGTCATCTCTGG |
| IL1B | H | FORWARD | CACGATGCACCTGTACGA |
|  |  | REVERSE | AACACCACTTGTTGCTCCA |
| IL6 | H | FORWARD | TGTGTGAAAGCAGCAAAGA |
|  |  | REVERSE | ACCAGGCAAGTCTCCTCA |

**Table S3.** Primers related to macrophage polarization.

| Primer name | Species | Orientation | Primer sequence (5’-3’) |
| --- | --- | --- | --- |
| Actb | M | FORWARD | TCTTTGCAGCTCCTTCGT |
|  |  | REVERSE | GACCCATTCCCACCATC |
| Tnf | M | FORWARD | CGCTGAGGTCAATCTGC |
|  |  | REVERSE | GGCTGGGTAGAGAATGGA |
| Nos2 | M | FORWARD | ACTCAGCCAAGCCCTCA |
|  |  | REVERSE | CTCTGCCTATCCGTCTCGT |
| Mrc1 | M | FORWARD | TGGCAAGTATCCACAGCA |
|  |  | REVERSE | GGTTCCATCACTCCACTCA |
| Il4 | M | FORWARD | TGTAGGGCTTCCAAGGTG |
|  |  | REVERSE | GAATCCAGGCATCGAAAA |
